# Association of History of Psychopathology With Accelerated Aging at Midlife

**DOI:** 10.1001/jamapsychiatry.2020.4626

**Published:** 2021-02-17

**Authors:** Jasmin Wertz, Avshalom Caspi, Antony Ambler, Jonathan Broadbent, Robert J. Hancox, HonaLee Harrington, Sean Hogan, Renate M. Houts, Joan H. Leung, Richie Poulton, Suzanne C. Purdy, Sandhya Ramrakha, Line Jee Hartmann Rasmussen, Leah S. Richmond-Rakerd, Peter R. Thorne, Graham A. Wilson, Terrie E. Moffitt

**Affiliations:** 1 Department of Psychology and Neuroscience, Duke University, Durham, North Carolina; 2 Institute of Psychiatry, Psychology & Neuroscience, King’s College London, London, United Kingdom; 3 Center for Genomic and Computational Biology, Duke University, Durham, North Carolina; 4 Department of Psychiatry and Behavioral Sciences, Duke University, Durham, North Carolina; 5 Promenta Research Center, University of Oslo, Norway; 6 Department of Psychology, University of Otago, Dunedin, New Zealand; 7 Department of Oral Sciences, University of Otago, Dunedin, New Zealand; 8 Department of Preventive & Social Medicine, University of Otago, Dunedin, New Zealand; 9 School of Psychology, University of Auckland, Auckland, New Zealand; 10 Centre for Brain Research, University of Auckland, Auckland, New Zealand; 11 Eisdell Moore Centre for Hearing and Balance Research, University of Auckland, Auckland, New Zealand; 12 Department of Clinical Research, Copenhagen University Hospital, Hvidovre, Denmark; 13 Department of Psychology, University of Michigan, Ann Arbor

## Abstract

**Importance:**

Individuals with mental disorders are at an elevated risk of developing chronic age-related physical diseases. However, it is not clear whether psychopathology is also associated with processes of accelerated aging that precede the onset of age-related disease.

**Objective:**

To test the hypothesis that a history of psychopathology is associated with indicators of accelerated aging at midlife.

**Design, Setting, and Participants:**

This prospective cohort study was based on the Dunedin Multidisciplinary Health and Development Study, a population-representative birth cohort of 1037 individuals born between April 1, 1972, and March 31, 1973, in Dunedin, New Zealand. Members were followed up to age 45 years (until April 2019). Data were analyzed from January 6 to December 7, 2020.

**Exposures:**

Mental disorders were assessed in 6 diagnostic assessments from ages 18 to 45 years and transformed through confirmatory factor analysis into continuous measures of general psychopathology (p-factor) and dimensions of internalizing, externalizing, and thought disorders (all standardized to a mean [SD] of 100 [15]).

**Main Outcomes and Measures:**

Signs of aging (biological pace of aging; declines in sensory, motor, and cognitive functioning; and facial age) were assessed up to age 45 years using previously validated measures including biomarkers, clinical tests, and self-reports.

**Results:**

Of the original 1037 cohort participants, 997 were still alive at age 45 years, of whom 938 (94%) were assessed (474 men [50.5%]). Participants who had experienced more psychopathology exhibited a faster pace of biological aging (β, 0.27; 95% CI, 0.21-0.33; *P* < .01); experienced more difficulties with hearing (β, 0.18; 95% CI, 0.12-0.24; *P* < .01), vision (β, 0.08; 95% CI, 0.01-0.14; *P* < .05), balance (β, 0.20; 95% CI, 0.14-0.26; *P* < .01), and motor functioning (β, 0.19; 95% CI, 0.12-0.25; *P* < .01); experienced more cognitive difficulties (β, 0.24; 95% CI, 0.18-0.31; *P* < .01); and were rated as looking older (β, 0.20; 95% CI, 0.14-0.26; *P* < .01). Associations persisted after controlling for sex, childhood health indicators, maltreatment, and socioeconomic status and after taking into account being overweight, smoking, use of antipsychotic medication, and the presence of physical disease. Tests of diagnostic specificity revealed that associations were generalizable across externalizing, internalizing, and thought disorders.

**Conclusions and Relevance:**

In this cohort study, a history of psychopathology was associated with accelerated aging at midlife, years before the typical onset of age-related diseases. This link is not specific to any particular disorder family but generalizes across disorders. Prevention of psychopathology and monitoring of individuals with mental disorders for signs of accelerated aging may have the potential to reduce health inequalities and extend healthy lives.

## Key Points

**Question:**

Is psychopathology associated with accelerated aging at midlife?

**Findings:**

In this population-representative birth cohort study of 1037 individuals followed up to age 45 years, a history of psychopathology was associated with a faster pace of biological aging; declines in sensory, motor, and cognitive functioning; and being rated as looking older. Associations persisted after controlling for sex, childhood health, maltreatment, and socioeconomic status and after taking into account being overweight, smoking, using antipsychotic medication, and having a physical disease; associations generalized across externalizing, internalizing, and thought disorders.

**Meaning:**

Results suggest that the prevention of psychopathology and monitoring of individuals with mental disorders for signs of accelerated aging may have the potential to reduce health inequalities and extend healthy lives.

## Teaser

This cohort study uses data from the Dunedin Multidisciplinary Health and Development Study to investigate whether psychopathology is associated with the processes of accelerated aging.

## Introduction

Individuals with mental disorders have a reduced life expectancy by approximately 10 to 20 years.^1,2,3^ Earlier mortality is only partly explained by unnatural causes of death, such as suicide.^1^ The bulk of the mortality gap is attributable to deaths from physical disease, including chronic age-related diseases such as cardiovascular disease, diabetes, and cancer.^1,2^ A major driver of these diseases is the process of aging itself.^4^ Mental disorders tend to onset and peak in the first 3 decades of life, whereas physical diseases onset and peak decades later, suggesting that processes of biological aging in the interim connect them.^5^ Here, we present an initial test of the hypothesis that psychopathology is associated with faster aging, that is, with accelerated physiological decline toward age-related disease and mortality. We tested this hypothesis in a population-representative cohort of New Zealanders who have been followed up from birth to midlife.^6^

The hypothesis that psychopathology is associated with accelerated aging is based on research that documents a connection between mental disorders and age-related medical conditions. Cross-sectional studies have reported comorbidity between mental disorders and physical diseases.^7^ Studies analyzing retrospective self-reports suggest that mental disorders precede physical diseases.^8^ Studies using administrative registry data document that official diagnoses of mental disorders predict diagnoses of physical diseases.^9^ Whereas past research has tended to focus on links between specific mental disorders and specific physical diseases (eg, schizophrenia and diabetes or depression and coronary heart disease),^10,11^ recent findings suggest that mental disorders are associated with multiple age-related diseases.^9,12^ This finding of nonspecific risk is consistent with geroscience theory positing that all age-related diseases share a common cause, which is aging itself.^4^ Mental disorders are associated with a variety of late-life physical diseases, which suggests the hypothesis that mental disorders are a precursor of accelerated aging.^5^ Although it might seem early to assess aging at midlife, recent work has shown that accelerated aging can already be quantified at this stage of life and that it forecasts earlier onset of future age-related physical diseases.^13,14^

The methodologic desiderata for testing the aging hypothesis are as follows: a longitudinal design, prospective assessments of multiple mental disorders, a population-based sample, and a new focus on measuring indicators of aging per se rather than physical disease. The present study combines these features to test associations between mental disorders and aging. To capture psychopathology, we measured symptoms of multiple externalizing, internalizing, and thought disorders using information from repeated diagnostic assessments between the ages of 18 to 45 years. To capture signs of aging, we evaluated the coordinated, progressive loss of integrity across cardiovascular, metabolic, pulmonary, kidney, immune, and dental systems using a biomarker composite of pace of aging that has previously been developed and validated.^15^ This measure captures correlated gradual decline across organ systems and reflects differences among individuals in their rate of biological aging by midlife. In addition to the pace of aging, we also measured sensory, motor, and cognitive functioning at midlife using assessments that are typically used in gerontology. Each aging outcome was assessed with a laboratory test or clinical assessment as well as a paired self-report. We used both laboratory tests and self-reports because these measures complement each other: laboratory tests capture objective levels of functioning that can be compared to normative levels, whereas self-reports capture participants’ own perception of functioning and impairment in everyday life. We also measured observer ratings of participants’ facial age.

Individuals who experience psychopathology and accelerated aging might have been in worse health already as children. To our knowledge, no prior studies have addressed this potential confound. Longitudinal data are needed to establish an early-life health baseline against which to compare associations between psychopathology and aging. There is also a need to rule out confounding by adverse early exposures, such as maltreatment or poverty.^16^ We used prospective measures of childhood health, maltreatment, and family socioeconomic status to account for these factors. In addition, we tested whether associations were limited to overweight individuals, smokers, those on antipsychotic medication whose side effects may mimic signs of accelerated aging,^17^ and individuals who had already been diagnosed with an age-related disease.

## Methods

### Sample

Participants were members of the Dunedin Multidisciplinary Health and Development Study (Dunedin Study). The full cohort comprises all individuals born between April 1, 1972, and March 31, 1973, in Dunedin, New Zealand, who were eligible based on residence in the province and who participated in the first assessment at age 3 years.^18^ The cohort represents the full range of socioeconomic status in the general population of New Zealand’s South Island. On adult health, the cohort matches the New Zealand National Health and Nutrition Survey on key health indicators (eg, body mass index [BMI], smoking, and visits to a physician).^18^ The cohort consists of primarily White participants (93%), matching South Island demographics. Assessments were carried out at birth and ages 3, 5, 7, 9, 11, 13, 15, 18, 21, 26, 32, 38, and, most recently, 45 years, when 938 of the 997 living cohort members (94%) took part (completed April 2019). Written informed consent was obtained from all participants at each assessment. Study protocols were approved by the institutional ethical review boards of the participating universities. The present study, based on the population-representative Dunedin birth cohort, was conducted from January 6 to December 7, 2020. This study followed the Strengthening the Reporting of Observational Studies in Epidemiology (STROBE) reporting guideline.

### Assessment of Mental Disorders

The Dunedin Study longitudinally ascertains mental disorders every few years, interviewing members about past-year symptoms (eAppendix 1 in the Supplement). Private structured interviews were conducted by health professionals blinded to participants’ prior data using the Diagnostic Interview Schedule^19^ at ages 18, 21, 26, 32, 38, and 45 years. We studied *DSM*–defined symptoms of 14 disorders: externalizing disorders (attention-deficit/hyperactivity disorder, conduct disorder, alcohol dependence, tobacco dependence, cannabis dependence, and other drug dependence), internalizing disorders (generalized anxiety disorder, depression, fears [including social phobia, simple phobia, agoraphobia, and panic disorder], eating disorders [including bulimia and anorexia], and posttraumatic stress disorder), and thought disorders (obsessive-compulsive disorder, mania, and schizophrenia) (eFigure 1 in the Supplement). Ordinal measures represented the number of possible *DSM*–defined symptoms associated with each disorder. Past-year prevalence rates of mental disorders in the Dunedin Study cohort are similar to prevalence rates in nationwide surveys of the US and New Zealand.^20^

The method used to evaluate the structure of psychopathology in the Dunedin Study cohort has been described previously^6,21^ (eAppendix 2 in the Supplement). Briefly, confirmatory factor analysis at the symptom level was used to test 2 standard models^21^: (1) a correlated-factors model and (2) a hierarchical or bifactor model. Using a correlated-factors model (eFigure 2 in the Supplement), 3 factors were tested, representing externalizing (with loadings from attention-deficit/hyperactivity disorder, conduct disorder, and alcohol, cannabis, tobacco, and other drug dependence), internalizing (with loadings from depression, generalized anxiety disorder, fears/phobias, posttraumatic stress disorder, and eating disorders), and thought disorders (with loadings from obsessive-compulsive disorder, mania, and schizophrenia). The model fit the data well (eTable 1 in the Supplement), confirming that 3 correlated factors (ie, internalizing, externalizing, and thought disorder) explain well the structure of the disorder symptoms. A hierarchical or bifactor model (eFigure 2 in the Supplement) established that symptom measures reflect both general psychopathology and narrower styles of psychopathology. General psychopathology (labeled p-factor) is represented by a factor that directly influences all of the diagnostic symptom factors. This model fit the data well (eTable 1 in the Supplement). The p-factor captures how cohort members differ from each other in the variety and persistence of many different kinds of symptoms from ages 18 to 45 years. Previous work has shown that Dunedin Study participants with higher p-factor scores experienced a younger age at onset, greater number of assessment ages with a disorder, and greater diversity of diagnoses (*r* = 0.76; 95% CI, 0.74-0.79) (eFigure 3 in the Supplement).^6^

### Measuring Signs of Accelerated Aging in Midlife

We measured participants’ pace of aging, integrity of sensory and motor systems (hearing, vision, balance, and motor functioning), and cognitive functioning by using previously validated measures based on assessments typical of gerontology (Table 1 and eAppendix 3 in the Supplement). Each aging outcome was measured using a laboratory test (eg, biomarkers; hearing test) as well as a related self-report (eg, of self-reported perceived age; hearing difficulties). Laboratory tests and self-reports were modestly correlated (eTable 2 in the Supplement), indicating that these measures assess related but different aspects of functioning. All measures were coded so that higher scores indicated greater difficulties.

**Table 1.** Description of Measures of Aging in Midlife and Matched Measures of Health in Childhood

Outcome	Type of measure	Description^a^	Ages, y
Pace of aging	Laboratory test	Participants’ pace of biological aging across ages 26-45 y was measured as changes in 19 biomarkers of cohort members’ cardiovascular, metabolic, pulmonary, kidney, immune, and dental systems across ages 26, 32, 38, and 45 y. The measure quantifies participants’ rate of aging in year-equivalent units of physiological decline per chronological year. The average participant experienced 1 y of physiological decline per year, a mean (SD) pace of aging of 1 (0.3).^13^	26-45
	Self-report	Participants’ self-perceived age at age 45 y was measured using participants’ response to the question, “Many people feel younger or older than they really are. What age do you feel most of the time?” The mean (SD) self-perceived age in participants aged 45 y was 40 (8) years.	45
	Matched childhood measure	Participants’ childhood physical health between birth and age 11 was measured using birth records, medical exams, anthropometry, lung function testing, nurse ratings, and interviews with parents about health conditions (eg, asthma, childhood diabetes).^22^	Birth, 3, 5, 7, 9, 11
Hearing	Laboratory test	Participants’ social hearing (ability to hear in noisy environments) at age 45 y was measured using the Listening in Spatialised Noise–Sentences Test (LISN-S). The test determines speech reception thresholds for sentences presented in competing speech under various conditions. For our primary analyses, we used a measure of low cue speech reception threshold, reflecting hearing performance when the person is not receiving optimum auditory information.^23^	45
	Self-report	Participants’ hearing difficulties at age 45 y were measured using 3 items from the Speech, Spatial, and Qualities of Hearing Scale (SSQ12) (eg, “Can you follow the conversation in a busy restaurant?”).^24^	45
	Matched childhood measure	Participants’ childhood social hearing at age 11 was assessed using a speech-in-noise test. The test measures children’s ability to correctly identify words under conditions of no noise, 10-, and 5-dB signal-to-noise ratio. To match the childhood measure to the adult hearing measure, we used children’s performance in the 5-dB signal-to-noise ratio condition, reflecting social hearing ability in the most difficult auditory environment.^25^	11
Vision	Laboratory test	Participants’ contrast sensitivity at age 45 y was measured using a Pelli-Robson chart administered by trained technicians. The chart presents 3 letters per line and the letters gradually fade from black to gray to white on a white background to determine the lowest level of “contrast” that the eye can detect. The resulting measure is a contrast sensitivity score function, reflecting a person’s best-corrected contrast detection threshold, the lowest contrast at which a pattern can be seen.	45
	Self-report	Participants’ vision difficulties at age 45 y were assessed using the 10-item Vision Quality of Life Core Measure (VCM1) questionnaire (eg, “How often has your eyesight stopped you from doing the things you wanted to do?”).^26^	45
	Matched childhood measure	Participants’ childhood vision was assessed at ages 7, 9, and 11 y using the Sheridan Gardiner single optotype letter matching test at 6 m (at age 7 y) and a 4-m logarithmic test chart (at ages 9 and 11 y).^27^	7, 9, 11
Balance	Laboratory test	Participants’ balance at age 45 y was measured using the Unipedal Stance Test, as the maximum time achieved across 3 trials of the test with eyes closed.^28^	45
	Self-report	Participants’ balance difficulties at age 45 y were measured using 4 items (eg, “Do you have feelings that things are spinning or moving around?”).	45
	Matched childhood measure	Participants’ childhood balance at ages 3, 7, and 9 y was measured using the balance subtests of the Bayley Motor Scales (age 3 y) and of the Basic Motor Ability Test (ages 7 and 9 y).^29,30^	3, 7, 9
Motor function	Laboratory test	Participants’ gait speed (m/s) at age 45 y was measured using the GAITRite Electronic Walkway (CIR Systems Inc). Gait speed was assessed under 3 conditions: usual gait speed (walk at normal pace; mean of 2 walks) and 2 challenge paradigms, dual task gait speed (walk at normal pace while reciting alternate letters of the alphabet out loud; mean of 2 walks), and maximum gait speed (walk as fast as safely possible; mean of 3 walks). We calculated the mean of the 3 individual walk conditions to generate a measure of composite gait speed.^13^	45
	Self-report	Participants’ physical function at age 45 y was assessed using the 10-item RAND 36-Item Health Survey 1.0 physical functioning scale, with reversed scores to reflect limitations.^31^	45
	Matched childhood measure	Participants’ childhood motor development at ages 3, 5, 7, and 9 y was measured using the Bayley Motor Scales (age 3 y), McCarthy Motor Scales (age 5 y) and Basic Motor Ability Test (ages 7 and 9 y).^29,30,32^	3, 5, 7, 9
Cognitive function	Laboratory test	Participants’ cognitive ability at age 45 y was measured using the Wechsler Adult Intelligence Scale–IV (WAIS-IV), individually administered.^33^	45
	Self-report	Participants’ cognitive complaints at age 45 y were measured using 23 items mapping onto *DSM-5* symptoms of mild neurocognitive disorder and items from the Cognitive Failures Questionnaire (CFQ), eg, “I have difficulty finding the word I want to use”; “I repeat myself, I tell the same story to the same person”; “I forget why I went from one part of the house to the other.”^34,35^	45
	Matched childhood measure	Participants’ childhood cognitive ability at ages 7, 9, and 11 y was measured using the Wechsler Intelligence Scale for Children – Revised (WISC-R), individually administered at each age. IQ scores for the 3 ages were averaged.^36^	7, 9, 11

^a^ More information is available in eAppendix 3 in the Supplement.

### Measuring Childhood Health Indicators, Maltreatment, and Socioeconomic Status

We matched prospectively collected childhood measures to each of the midlife outcomes (eg, childhood hearing test was matched to midlife hearing; childhood motor test to midlife motor functioning; and childhood cognitive test to midlife cognitive functioning) (Table 1 and eAppendix 3 in the Supplement). Correlations between childhood and midlife measures ranged from 0.11 (vision) to 0.78 (cognitive function) for laboratory tests at midlife and from 0.05 (balance) to 0.20 (motor skills) for self-reports at midlife (eTable 3 in the Supplement). Childhood maltreatment and childhood socioeconomic status were measured as previously described (eAppendix 4 in the Supplement).^16,37^

### Measuring BMI, Smoking, Antipsychotic Medication Use, and Medical Disease

Participants’ BMI (calculated as weight in kilograms divided by height in meters squared) was recorded at the participants’ assessment at age 45 years. Smoking was coded as whether participants had reported daily smoking at any assessment up to age 45 years (478 of 926 reporting participants [51.6%]). Antipsychotic medication use at age 45 years was assessed in standardized interviews about their medications, and participants brought medications on the assessment day, which were evaluated by a pharmacist. Antipsychotics were used by 18 of 938 reporting participants (1.9%). Cancer or heart attack by age 45 years was assessed by standardized interviews that ascertained whether participants had been told by a health professional that they had any of several diseases. Diabetes was assessed based on participants’ blood levels of glycated hemoglobin.^15^ In line with clinical diagnostic criteria, a cutoff of 48 mmol/mol was used. Cancer, heart attacks, or diabetes by age 45 years affected 58 of 932 reporting participants (6.2%).

### Statistical Analysis

We performed ordinary least squares regression to test whether a history of psychopathology was associated with signs of accelerated aging at midlife. To test whether childhood health indicators could explain these associations, we controlled for childhood measures matched to each of the midlife outcomes. To test whether childhood socioeconomic status or maltreatment could explain associations, we controlled for these measures. In sensitivity analyses, BMI and smoking status up to age 45 years were added as covariates, and analyses were repeated after excluding individuals taking antipsychotic medications or those who had cancer, a heart attack, or diabetes. All analyses were adjusted for participants’ sex. We present standardized regression coefficients (β) for all associations. Statistical analyses were performed in Mplus, version 7.2 using full information maximum likelihood.^38^ Statistical analyses were performed from January 6 to December 7, 2020. Results reported here were checked for reproducibility by an independent data analyst (R.M.H.), who recreated the code by working from the manuscript and applying it to a fresh copy of the data set. All *P* values were 2-sided, and *P* < .05 was considered statistically significant.

## Results

Of the original 1037 cohort participants, 997 were still alive at age 45 years; of these, 938 (94%) participated in the assessment at age 45 years (474 men [50.5%]). Participants seen at age 45 years did not differ significantly from other living participants in terms of childhood socioeconomic status, childhood intelligence quotient, childhood physical health, or p-factor scores (eAppendix 5 in the Supplement).

### Associations Between a History of Psychopathology and Laboratory Tests of Aging at Midlife

**Figure 1.**
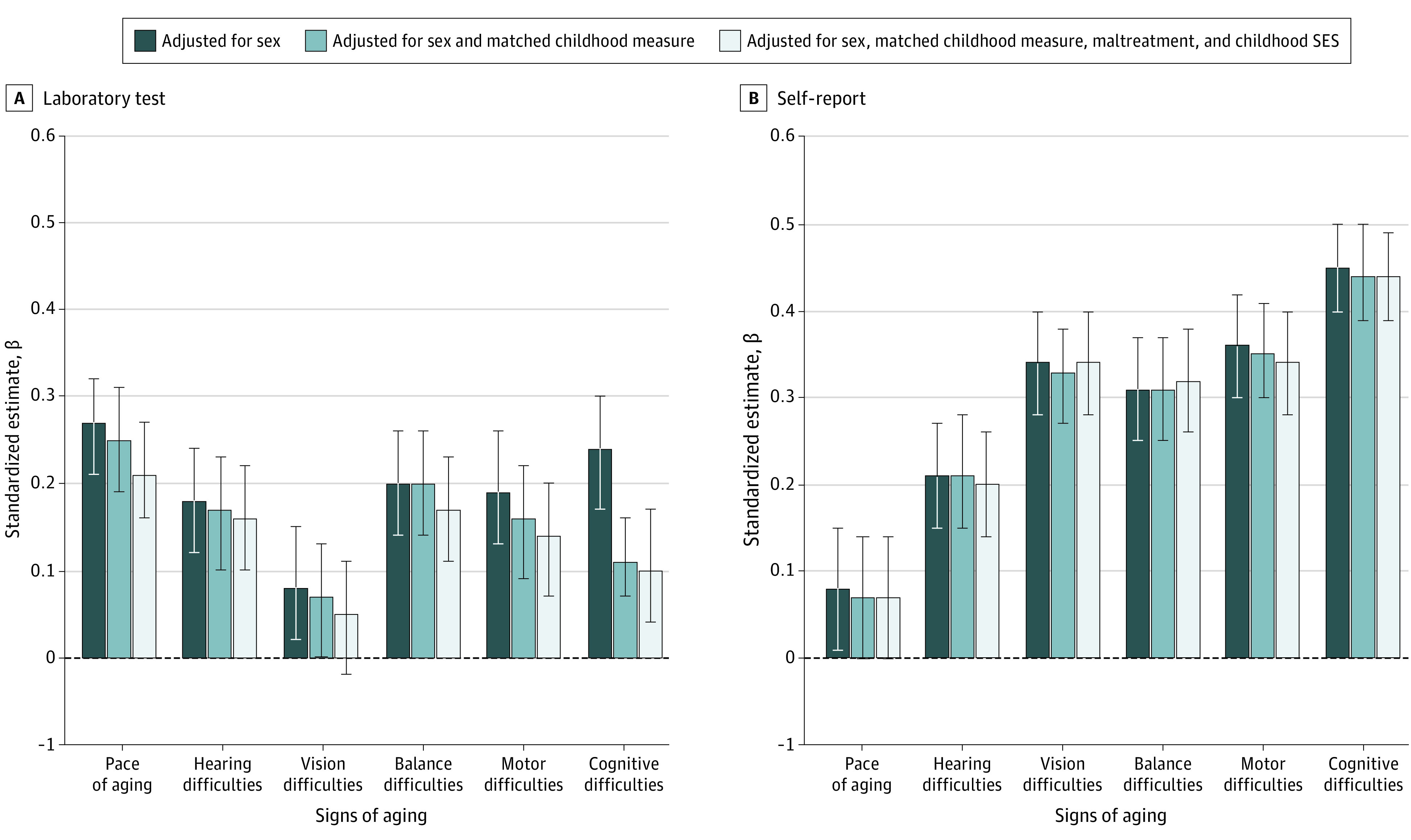
Associations Between Participants’ Psychopathology p-Factor Scores and Signs of Aging at Midlife Participants’ p-factor scores were measured as a latent factor summarizing the common variance among all of the multiple psychiatric symptoms ever experienced by participants between ages 18 and 38 years. A, Effect sizes of associations between p-factor scores and signs of aging measured using laboratory tests. Outcome measures are coded so that higher scores indicate a worse outcome (eg, worse hearing, worse motor skills, and lower intelligence quotient). Error bars indicate 95% CIs. A childhood measure was matched to each adult outcome (eg, childhood hearing test to adult hearing test, childhood motor test to adult motor test, and childhood cognitive test to adult cognitive test). B, Effect sizes of associations between p-factor scores and signs of aging measured using self-reports. Outcome measures are coded so that higher scores indicate a worse outcome (eg, worse hearing, worse motor skills, and more cognitive difficulties). Error bars indicate 95% CIs. SES indicates socioeconomic status.

**Figure 2.**
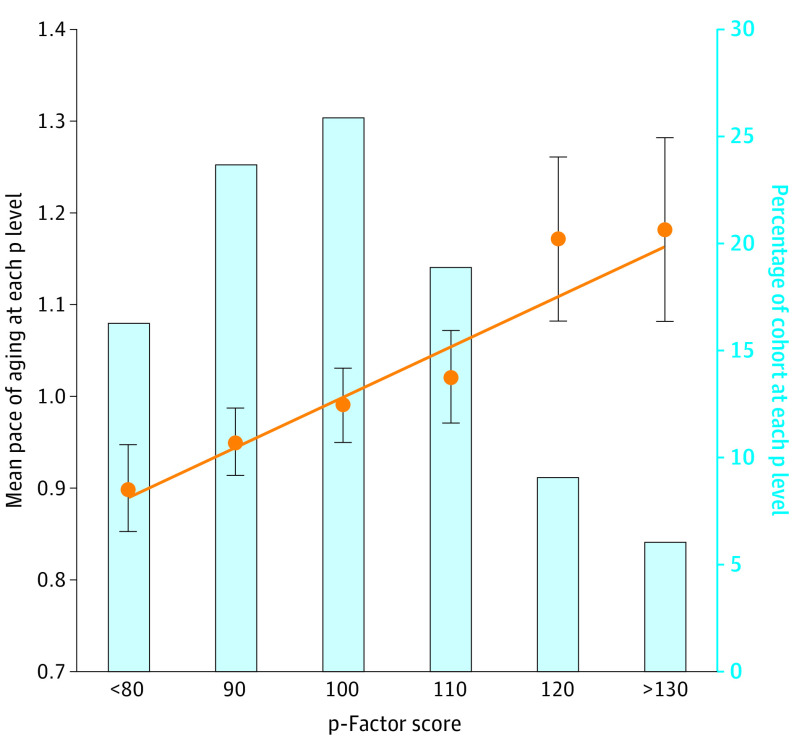
Associations Between Dunedin Study Participants’ Psychopathology p-Factor Scores and Pace of Aging Participants’ p-factor scores (mean [SD], 100 [15]) were measured as a latent factor summarizing the common variance among all of the multiple psychiatric symptoms ever experienced by participants between ages 18 and 45 years. Participants were placed into 10-point bins based on their p-factor scores for graphing purposes only. The cutoffs are the midpoint of each category, ie, up to 85, up to 95, up to 105, up to 115, up to 125, and 125 and above. Pace of aging was measured from changes in 19 biomarkers of participants’ cardiovascular, metabolic, pulmonary, kidney, immune, and dental systems when participants were aged 26, 32, 38, and 45 years. Error bars indicate 95% CIs. The regression line shows the correlation between p-factor and pace of aging (β, 0.27; 95% CI, 0.21-0.33; *P* < .01), calculated on the full score distribution.

Participants with higher scores on a general factor of psychopathology (p-factor) covering symptoms from ages 18 to 45 years were aging faster at midlife, as evident across all signs of aging (Figure 1A). Analyses of pace of aging showed that participants with higher p-factor scores exhibited a faster pace of biological aging between ages 26 and 45 years (β, 0.27; 95% CI, 0.21-0.33; *P* < .01) (Figure 1A). This association amounted to approximately 5.3 more years of biological aging for participants with the highest vs lowest p-factor scores (Figure 2). Analyses of sensory and motor functioning showed that participants with higher p-factor scores experienced lower integrity of sensory and motor systems at age 45 years, including more difficulties with social hearing (β, 0.18; 95% CI, 0.12-0.24; *P* < .01), vision (β, 0.08; 95% CI, 0.01-0.14; *P* < .05), balance (β, 0.20; 95% CI, 0.14-0.26; *P* < .01), and gait speed (β, 0.19; 95% CI, 0.12-0.25; *P* < .01) (Figure 1A). Analyses of cognitive functioning showed that participants with higher p-factor scores experienced more cognitive difficulties at age 45 years (β, 0.24; 95% CI, 0.18-0.31; *P* < .01) (Figure 1A).

Individuals who experienced psychopathology and signs of accelerated aging by age 45 years might have had a history of poor childhood health. Indeed, childhood measures matched to each outcome in adulthood (eg, childhood hearing test to adult hearing test, childhood motor test to adult motor test, and childhood cognitive test to adult cognitive test) preceded both psychopathology and faster aging in adulthood (hearing, β = 0.12 [95% CI, 0.04-0.19] for psychopathology vs 0.14 [95% CI, 0.07-0.22] for self-reported accelerated aging; motor skills, β = 0.11 [95% CI, 0.04-0.17] for psychopathology vs 0.20 [95% CI, 0.13-0.27] for self-reported accelerated aging; and cognitive function, β = 0.18 [95% CI, 0.12-0.25] for psychopathology vs 0.18 [95% CI, 0.11-0.24] for self-reported accelerated aging) (eTable 3 in the Supplement). However, psychopathology continued to be associated with most outcomes after controlling for childhood measures, indicating that a history of psychopathology was associated with greater declines in physiological, sensory, motor, and cognitive functioning when measured against an individual’s baseline of poor health in childhood (pace of aging, β = 0.21 [95% CI, 0.15-0.27]; hearing, β = 0.16 [95% CI, 0.09-0.22]; vision, β = 0.05 [95% CI, –0.02 to 0.11]; balance, β = 0.17 [95% CI, 0.11-0.23]; motor, β = 0.14 [95% CI, 0.08-0.20]; and cognitive, β = 0.10 [95% CI, 0.05-0.14]) (Table 2 and Figure 1A). For example, individuals with higher p-factor scores had significantly slower gait speed at midlife, even after controlling for preexisting childhood motor difficulties, and they performed more poorly on tests of cognition, even after controlling for their preexisting childhood cognitive abilities (ie, they experienced greater measured child-to-midlife cognitive decline, not just low cognitive functioning present since childhood).

**Table 2.** Associations Between Participants’ p-Factor; Externalizing, Internalizing, and Thought Disorder Scores; and Signs of Aging at Midlife, Controlling for Sex, Childhood Health Indicators, Childhood Maltreatment, and Childhood Socioeconomic Status^a^^,^^b^

Sign of aging	β (95% CI)^c^				No.^d^
	p-Factor score	Externalizing disorder score	Internalizing disorder score	Thought disorder score	
Laboratory test					
Pace of aging	0.21 (0.15 to 0.27)	0.21 (0.15 to 0.27)	0.18 (0.11 to 0.24)	0.21 (0.15 to 0.27)	932
Hearing difficulties	0.16 (0.09 to 0.22)	0.13 (0.06 to 0.20)	0.15 (0.08 to 0.21)	0.15 (0.09 to 0.22)	902
Vision difficulties	0.05 (–0.02 to 0.11)	–0.02 (–0.09 to 0.05)	0.04 (–0.03 to 0.11)	0.03 (–0.03 to 0.10)	904
Balance difficulties	0.17 (0.11 to 0.23)	0.12 (0.06 to 0.19)	0.15 (0.08 to 0.21)	0.16 (0.10 to 0.22)	911
Motor difficulties	0.14 (0.08 to 0.20)	0.08 (0.02 to 0.15)	0.15 (0.09 to 0.22)	0.14 (0.08 to 0.21)	904
Cognitive difficulties	0.10 (0.05 to 0.14)	0.13 (0.08 to 0.17)	0.06 (0.02 to 0.11)	0.10 (0.05 to 0.14)	918
Self-report					
Pace of aging	0.07 (0.00 to 0.14)	0.00 (–0.07 to 0.06)	0.07 (0.00 to 0.14)	0.06 (0.00 to 0.13)	893
Hearing difficulties	0.20 (0.14 to 0.26)	0.09 (0.03 to 0.16)	0.18 (0.11 to 0.23)	0.18 (0.11 to 0.24)	924
Vision difficulties	0.34 (0.28 to 0.39)	0.18 (0.11 to 0.24)	0.32 (0.26 to 0.38)	0.32 (0.26 to 0.38)	925
Balance difficulties	0.32 (0.26 to 0.38)	0.17 (0.11 to 0.24)	0.28 (0.22 to 0.34)	0.30 (0.24 to 0.35)	924
Motor difficulties	0.34 (0.28 to 0.40)	0.20 (0.13 to 0.26)	0.30 (0.24 to 0.37)	0.31 (0.25 to 0.37)	923
Cognitive difficulties	0.44 (0.38 to 0.49)	0.25 (0.18 to 0.31)	0.36 (0.30 to 0.42)	0.38 (0.33 to 0.44)	917

^a^ Participants’ p-factor scores were measured as a latent factor summarizing the common variance among all of the multiple psychiatric symptoms ever experienced by participants between ages 18 and 45 years. Internalizing, externalizing, and thought disorder factor scores were measured as correlated latent factors summarizing common variance among internalizing, externalizing, and thought disorder symptoms ever experienced by participants between ages 18 and 45 years.

^b^ All associations are adjusted for participants’ sex, a childhood measure matched to each adult outcome (eg, childhood hearing test to adult hearing test, childhood motor test to adult motor test, and childhood cognitive test to adult cognitive test), childhood maltreatment, and childhood socioeconomic status.

^c^ β Indicates standardized estimate.

^d^ A total of 938 participants were assessed at age 45 years (94% of the living Dunedin Study participants). The No. column reflects participants who have both psychopathology data and age-45-years outcome data.

Associations also remained statistically significant after controlling for childhood maltreatment and childhood socioeconomic status (Figure 1A). Sensitivity analyses further showed that some associations reduced but were not explained away when including BMI and smoking in the models. These associations were reduced by approximately 6% for BMI (associations reduced by approximately 6% for BMI and 20% for smoking; eTable 4 in the Supplement), and they were not attributable to use of antipsychotic medication or to already being diagnosed with an age-related disease (eTable 4 in the Supplement).

### Associations Between Adult Mental Disorders and Self-reports of Aging at Midlife

Findings of associations between p-factor scores and laboratory tests of aging were corroborated by participants’ self-reports (Figure 1B). Participants with higher p-factor scores reported feeling significantly older than same-aged peers with lower p-factor scores (β, 0.08; 95% CI, 0.01-0.15; *P* = .02) (Figure 1B). Participants also reported greater difficulties with their social hearing (β, 0.21; 95% CI, 0.15-0.27; *P* < .01), vision (β, 0.34; 95% CI, 0.28-0.40; *P* < .01), balance (β, 0.31; 95% CI, 0.25-0.37; *P* < .01), and motor functioning (β, 0.36; 95% CI, 0.30-0.42; *P* < .01) (Figure 1B), as well as greater cognitive impairment, eg, having difficulty finding the words they want to use and forgetting appointments (β, 0.45; 95% CI, 0.40-0.50; *P* < .01) (Figure 1B). Controlling for matched childhood health measures, maltreatment and socioeconomic status did not change most findings (Table 2 and Figure 1B), nor were associations attributable to being overweight, smoking, use of antipsychotic medication, or age-related disease (eTable 4 in the Supplement).

### Testing Specificity in the Association Between Psychopathology and Accelerated Aging

**Figure 3.**
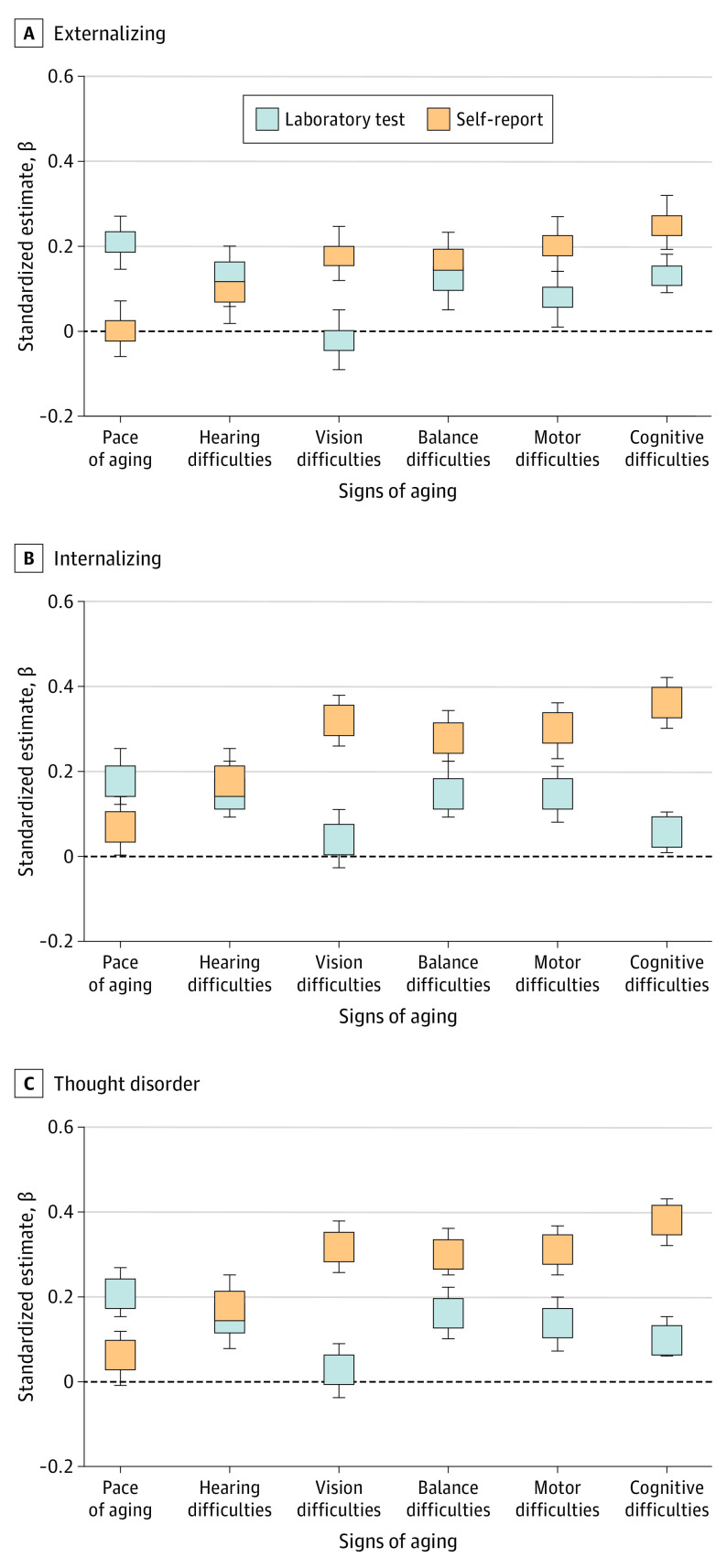
Associations Between Psychopathology and Signs of Accelerated Aging Separately for Externalizing, Internalizing, and Thought Disorder Families All associations are adjusted for participants’ sex, a childhood measure matched to each adult outcome (eg, childhood hearing test to adult hearing test, childhood motor test to adult motor test, and childhood cognitive test to adult cognitive test), childhood maltreatment, and childhood socioeconomic status. For associations between psychopathology and hearing difficulties, estimates overlapped between laboratory test and self-report. Error bars indicate 95% CIs.

The internalizing, externalizing, and thought disorder factors from the correlated-factor model were similarly associated with signs of aging, indicating that links between psychopathology and aging were not specific to 1 particular disorder family but generalizable across disorders (pace of aging: externalizing β, 0.21; 95% CI, 0.15-0.27; internalizing β, 0.18; 95% CI, 0.11-0.24; and thought β, 0.21; 95% CI, 0.15-0.27; hearing: externalizing β, 0.13; 95% CI, 0.06-0.20; internalizing β, 0.15; 95% CI, 0.08-0.21; and thought β, 0.15; 95% CI, 0.09-0.22; vision: externalizing β, –0.02; 95% CI, –0.09 to 0.05; internalizing β, 0.04; 95% CI, –0.03 to 0.11; and thought β, 0.03; 95% CI, –0.03 to 0.10; balance: externalizing β, 0.12; 95% CI, 0.06-0.19; internalizing β, 0.15; 95% CI, 0.08-0.21; and thought β, 0.16; 95% CI, 0.10-0.22; motor: externalizing β, 0.08; 95% CI, 0.02-0.15; internalizing β, 0.15; 95% CI, 0.09-0.22; and thought β, 0.14; 95% CI, 0.08-0.21; and cognitive: externalizing β, 0.13; 95% CI, 0.08-0.17; internalizing β, 0.06; 95% CI, 0.02-0.11; and thought β, 0.10; 95% CI, 0.05-0.14) (Table 2 and Figure 3, laboratory tests).

### Testing Associations With Informant Ratings of Facial Age

To summarize the link between psychopathology history and accelerated aging, we tested whether participants aged 45 years with higher p-factor scores were rated as looking older by independent observers who rated participants’ age based on facial photographs (eAppendix 3 in the Supplement). The results confirmed this association (β, 0.20; 95% CI, 0.14-0.26; *P* < .01). Associations were similar for each diagnostic family, indicating that links between mental disorders and facial aging generalize across externalizing (β, 0.22; 95% CI, 0.16-0.28; *P* < .01), internalizing (β, 0.16; 95% CI, 0.09-0.22; *P* < .01), and thought (β, 0.19; 95% CI, 0.13-0.26; *P* < .01) disorders.

## Discussion

Findings from this longitudinal cohort study suggest that individuals with younger age at mental disorder onset, a greater variety of mental disorders, and more persistence of mental disorders—as captured by higher scores on a general factor of psychopathology (p-factor)^6^—showed signs of accelerated aging already by midlife. Previous research findings suggest that mental disorders predict age-related physical disease.^8,9,12^ Here, we extend this research in 4 ways. First, rather than physical disease, we measured signs of aging itself, including accelerated biological aging; impairments in sensory, motor, and cognitive functioning; and facial age. Second, we found that the p-factor was associated with aging outcomes even when evaluated against a baseline of poor health in childhood, indicating that associations cannot be accounted for by environmental and/or genetic influences associated with poor health in early life. Third, we found that associations between mental disorders and aging were not specific to any 1 disorder family but generalized across externalizing, internalizing, and thought disorders. Fourth, sensitivity analyses revealed that associations were not explained by BMI, smoking, use of antipsychotic medication, or presence of age-related physical diseases. Taken together, our findings appear to support the hypothesis that psychopathology contributes to the cumulative, progressive, and gradual deterioration across organ systems that underlies biological aging.

Both self-report and laboratory test measures of aging were associated with psychopathology, although associations tended to be larger for self-reports. One possible explanation is that people with mental disorders provide a biased, pessimistic assessment of their health. Another possibility is that self-reports and laboratory tests capture different aspects of functioning (eg, the self-report vision measure is broader than the laboratory test, which captures only contrast sensitivity). Previous research suggests that self-reports and laboratory test measures independently predict later morbidity and mortality.^39^ Our findings indicate that both self-reports and laboratory test measures are associated with a history of mental disorders, suggesting that mental disorders may be an especially potent prevention and treatment target.

Our findings have implications for mental health research. The most prominent issue concerns mechanisms linking psychopathology to aging. Here, we articulate a research agenda and suggest research directions and testable hypotheses. First, associations between psychopathology and aging could arise because of poor health behaviors, such as smoking, lack of exercise, and poor diet.^40^ Indeed, many of the signs of aging measured in our study are influenced by health behaviors. Consistent with this possibility, associations were reduced when controlling for BMI and smoking, although they were not explained away. Second, individuals with mental disorders have reduced access to high-quality health care,^41^ which may contribute to associations with accelerated aging. Third, individuals with mental disorders are less likely to obtain educational qualifications and employment,^42^ increasing risk for poverty—a contributor to accelerated aging. Fourth, psychopathology is associated with stressful experiences, such as adverse childhood experiences and victimization in adulthood.^43^ In addition, experiencing mental disorder symptoms, such as anxiety, worry, paranoia, or shame, is itself stressful and often erodes one’s support network.^44^ Stress also increases the circulation of inflammatory and stress hormone biomarkers, which may connect psychopathology^45^ and age-related diseases.^46^ Fifth, genetic vulnerabilities might simultaneously increase an individual’s risk for psychopathology and faster aging, such as through shared genetic effects on mental disorders and risk factors for aging (eg, glucose dysregulation).^46^

Our findings have implications for health prevention and intervention. First, if the association between mental disorders and aging turns out to be causal, prevention of psychopathology has the potential to slow aging and delay age-related disease because mental disorders begin and reach their peak years earlier than physical diseases. This association presents an opportunity for psychiatry to reduce the burden of age-related disease and early mortality, which would benefit individuals with mental disorders as well as public health systems.^5,47^ Second, interventions could target possible intermediaries of associations between psychopathology and aging, ranging from reducing health-risk behaviors to fighting inflammation.^45^ Third, even if associations between psychopathology and aging are not causal, individuals with mental disorders are a high-priority group to monitor for signs of accelerated aging—such as hearing impairment, motor problems, and cognitive decline—that become apparent earlier in this group than in the general population. Such monitoring will require greater integration of mental and physical health services to reduce health inequalities and lengthen healthy lives.

### Limitations

Our study has limitations. First, measures of childhood health could not always be perfectly matched to adult outcomes (eg, we did not have a childhood test of gait speed to parallel the adult test, so we relied on results from comprehensive tests of children’s motor functioning). Second, participants have only been followed to age 45 years, so whether those with signs of accelerated aging will die younger is not yet known. However, other research has established that our outcome measures are associated with morbidity and mortality in older adults.^14,48^ Third, our measure of pace of aging was derived from 19 biomarkers repeatedly assessed over 20 years, which will be infeasible for most studies. However, we recently reported that a proxy measure can be quantified from methylation data extracted from a single cross-sectional blood draw,^14^ making it possible for studies lacking 4 waves of biomarkers to measure the pace of aging. Fourth, our observational design cannot rule out all possible confounders of associations between psychopathology and accelerated aging.

## Conclusions

The findings of this cohort study suggest that a history of psychopathology was associated with accelerated aging at midlife, years before the typical onset of age-related diseases. Furthermore, study results suggest that prevention of psychopathology and monitoring of individuals with mental disorders for signs of accelerated aging may have the potential to reduce health inequalities and extend healthy lives.

## Notes

Supplement. **eAppendix 1.** Measuring Psychopathology **eAppendix 2.** Modeling the Structure of Psychopathology **eAppendix 3.** Measuring Accelerated Aging **eAppendix 4.** Measuring Childhood Maltreatment and SES **eAppendix 5.** Attrition Analysis **eTable 1.** Standardized Factor Loadings for Models of the Structure of Psychopathology **eTable 2.** Correlations Between Laboratory/Clinical Test Measures and Self-Report Measures of Signs of Aging at Age 45 **eTable 3.** Association Between Childhood Health and Adult Psychopathology and Signs of Accelerated Aging **eTable 4.** Sensitivity Analyses of Associations Between P and Signs of Aging **eFigure 1.** Structure of Mental Disorder Data Collected in the Dunedin Study **eFigure 2.** Confirmatory Factor Analysis Models of the Structure of Psychopathology **eFigure 3.** Plot of the Positive Correlation Between the Variety and Persistence of Mental Disorders and P-Factor Scores in the Dunedin Study **eReferences**

## References

[1] Walker ER, McGee RE, Druss BG. Mortality in mental disorders and global disease burden implications: a systematic review and meta-analysis. JAMA Psychiatry. 2015;72(4):334-341. doi:10.1001/jamapsychiatry.2014.2502 25671328 PMC4461039

[2] Nordentoft M, Wahlbeck K, Hällgren J, . Excess mortality, causes of death and life expectancy in 270,770 patients with recent onset of mental disorders in Denmark, Finland and Sweden. PLoS One. 2013;8(1):e55176. doi:10.1371/journal.pone.0055176 23372832 PMC3555866

[3] Plana-Ripoll O, Pedersen CB, Agerbo E, . A comprehensive analysis of mortality-related health metrics associated with mental disorders: a nationwide, register-based cohort study. Lancet. 2019;394(10211):1827-1835. doi:10.1016/S0140-6736(19)32316-5 31668728

[4] Kennedy BK, Berger SL, Brunet A, . Geroscience: linking aging to chronic disease. Cell. 2014;159(4):709-713. doi:10.1016/j.cell.2014.10.039 25417146 PMC4852871

[5] Moffitt TE, Caspi A. Psychiatry’s opportunity to prevent the rising burden of age-related disease. JAMA Psychiatry. 2019;76(5):461-462. doi:10.1001/jamapsychiatry.2019.0037 30916735 PMC8327353

[6] Caspi A, Houts RM, Ambler A, . Longitudinal assessment of mental health disorders and comorbidities across 4 decades among participants in the Dunedin birth cohort study. JAMA Netw Open. 2020;3(4):e203221. doi:10.1001/jamanetworkopen.2020.3221 32315069 PMC7175086

[7] Buist-Bouwman MA, de Graaf R, Vollebergh WAM, Ormel J. Comorbidity of physical and mental disorders and the effect on work-loss days. Acta Psychiatr Scand. 2005;111(6):436-443. doi:10.1111/j.1600-0447.2005.00513.x 15877710

[8] Scott KM, Lim C, Al-Hamzawi A, . Association of mental disorders with subsequent chronic physical conditions: world mental health surveys from 17 countries. JAMA Psychiatry. 2016;73(2):150-158. doi:10.1001/jamapsychiatry.2015.2688 26719969 PMC5333921

[9] Momen NC, Plana-Ripoll O, Agerbo E, . Association between mental disorders and subsequent medical conditions. N Engl J Med. 2020;382(18):1721-1731. doi:10.1056/NEJMoa1915784 32348643 PMC7261506

[10] Rajan S, McKee M, Rangarajan S, ; Prospective Urban Rural Epidemiology (PURE) Study Investigators. Association of symptoms of depression with cardiovascular disease and mortality in low-, middle-, and high-income countries. JAMA Psychiatry. 2020;77(10):1052-1063. doi:10.1001/jamapsychiatry.2020.1351 32520341 PMC7287938

[11] Ward M, Druss B. The epidemiology of diabetes in psychotic disorders. Lancet Psychiatry. 2015;2(5):431-451. doi:10.1016/S2215-0366(15)00007-3 26360287

[12] Richmond-Rakerd LS, D’Souza S, Milne BJ, Caspi A, Moffitt TE. Longitudinal associations of mental disorders with physical diseases and mortality among 2.3 million New Zealanders. JAMA Netw Open. 2020;4(1):e2033448. doi:10.1001/jamanetworkopen.2020.33448 PMC7807295 33439264

[13] Rasmussen LJH, Caspi A, Ambler A, . Association of neurocognitive and physical function with gait speed in midlife. JAMA Netw Open. 2019;2(10):e1913123. doi:10.1001/jamanetworkopen.2019.13123 31603488 PMC6804027

[14] Belsky DW, Caspi A, Arseneault L, . Quantification of the pace of biological aging in humans through a blood test, the DunedinPoAm DNA methylation algorithm. Elife. 2020;9:e54870. doi:10.7554/eLife.54870 32367804 PMC7282814

[15] Belsky DW, Caspi A, Houts R, . Quantification of biological aging in young adults. Proc Natl Acad Sci U S A. 2015;112(30):E4104-E4110. doi:10.1073/pnas.1506264112 26150497 PMC4522793

[16] Poulton R, Caspi A, Milne BJ, . Association between children’s experience of socioeconomic disadvantage and adult health: a life-course study. Lancet. 2002;360(9346):1640-1645. doi:10.1016/S0140-6736(02)11602-3 12457787 PMC3752775

[17] Correll CU, Frederickson AM, Kane JM, Manu P. Metabolic syndrome and the risk of coronary heart disease in 367 patients treated with second-generation antipsychotic drugs. J Clin Psychiatry. 2006;67(4):575-583. doi:10.4088/JCP.v67n0408 16669722

[18] Poulton R, Moffitt TE, Silva PA. The Dunedin Multidisciplinary Health and Development Study: overview of the first 40 years, with an eye to the future. Soc Psychiatry Psychiatr Epidemiol. 2015;50(5):679-693. doi:10.1007/s00127-015-1048-8 25835958 PMC4412685

[19] Robins LN, Cottler L, Bucholz KK, Compton W. Diagnostic Interview Schedule for *DSM-IV*. Washington University School of Medicine; 1995.

[20] Schaefer JD, Caspi A, Belsky DW, . Enduring mental health: prevalence and prediction. J Abnorm Psychol. 2017;126(2):212-224. doi:10.1037/abn0000232 27929304 PMC5304549

[21] Caspi A, Houts RM, Belsky DW, . The p factor: one general psychopathology factor in the structure of psychiatric disorders? Clin Psychol Sci. 2014;2(2):119-137. doi:10.1177/2167702613497473 25360393 PMC4209412

[22] Belsky DW, Moffitt TE, Corcoran DL, . The genetics of success: how single-nucleotide polymorphisms associated with educational attainment relate to life-course development. Psychol Sci. 2016;27(7):957-972. doi:10.1177/0956797616643070 27251486 PMC4946990

[23] Cameron S, Dillon H. Development of the Listening in Spatialized Noise-Sentences Test (LISN-S). Ear Hear. 2007;28(2):196-211. doi:10.1097/AUD.0b013e318031267f 17496671

[24] Noble W, Jensen NS, Naylor G, Bhullar N, Akeroyd MA. A short form of the Speech, Spatial and Qualities of Hearing scale suitable for clinical use: the SSQ12. Int J Audiol. 2013;52(6):409-412. doi:10.3109/14992027.2013.781278 23651462 PMC3864780

[25] Welch D, Dawes PJD. Variation in the normal hearing threshold predicts childhood IQ, linguistic, and behavioral outcomes. Pediatr Res. 2007;61(6):737-744. doi:10.1203/pdr.0b013e31805341c1 17426656

[26] Frost NA, Sparrow JM, Durant JS, Donovan JL, Peters TJ, Brookes ST. Development of a questionnaire for measurement of vision-related quality of life. Ophthalmic Epidemiol. 1998;5(4):185-210. doi:10.1076/opep.5.4.185.4191 9894804

[27] Wilson GA, Welch D. Does amblyopia have a functional impact? findings from the Dunedin Multidisciplinary Health and Development Study. Clin Exp Ophthalmol. 2013;41(2):127-134. doi:10.1111/j.1442-9071.2012.02842.x 22712767

[28] Vereeck L, Wuyts F, Truijen S, Van de Heyning P. Clinical assessment of balance: normative data, and gender and age effects. Int J Audiol. 2008;47(2):67-75. doi:10.1080/14992020701689688 18236239

[29] Bayley N. The Bayley Scale of Infant Development. Psychological Corp; 1969.

[30] Arnheim DD, Sinclair WA. The Clumsy Child. VC Mosby Co; 1974.

[31] The RAND Corporation. RAND 36-Item Short Form Survey (SF-36). Accessed September 2020. https://www.rand.org/health-care/surveys_tools/mos/36-item-short-form.html

[32] McCarthy D. McCarthy Scales of Children’s Abilities. Psychological Corp; 1972.

[33] Wechsler D. Wechsler Adult Intelligence Scale: WAIS-IV: Technical and Interpretive Manual. Pearson; 2008.

[34] *American Psychiatric Association*. Diagnostic and Statistical Manual of Mental Disorders. 5th ed. American Psychiatric Association; 2013.

[35] Broadbent DE, Cooper PF, FitzGerald P, Parkes KR. The Cognitive Failures Questionnaire (CFQ) and its correlates. Br J Clin Psychol. 1982;21(1):1-16. doi:10.1111/j.2044-8260.1982.tb01421.x 7126941

[36] Wechsler D. Manual for the Wechsler Intelligence Scale for Children – Revised. Psychological Corporation; 1974.

[37] Caspi A, McClay J, Moffitt TE, . Role of genotype in the cycle of violence in maltreated children. Science. 2002;297(5582):851-854. doi:10.1126/science.1072290 12161658

[38] Muthén L, Muthén BO. *Mplus User’s Guide.* 7th ed. Muthén & Muthén; 2015.

[39] Idler EL, Benyamini Y. Self-rated health and mortality: a review of twenty-seven community studies. J Health Soc Behav. 1997;38(1):21-37. doi:10.2307/2955359 9097506

[40] Vermeulen-Smit E, Ten Have M, Van Laar M, De Graaf R. Clustering of health risk behaviours and the relationship with mental disorders. J Affect Disord. 2015;171:111-119. doi:10.1016/j.jad.2014.09.031 25303027

[41] Thornicroft G. Physical health disparities and mental illness: the scandal of premature mortality. Br J Psychiatry. 2011;199(6):441-442. doi:10.1192/bjp.bp.111.092718 22130744

[42] Dohrenwend BP, Levav I, Shrout PE, . Socioeconomic status and psychiatric disorders: the causation-selection issue. Science. 1992;255(5047):946-952. doi:10.1126/science.1546291 1546291

[43] Afifi TO, Enns MW, Cox BJ, Asmundson GJG, Stein MB, Sareen J. Population attributable fractions of psychiatric disorders and suicide ideation and attempts associated with adverse childhood experiences. Am J Public Health. 2008;98(5):946-952. doi:10.2105/AJPH.2007.120253 18381992 PMC2374808

[44] Uliaszek AA, Zinbarg RE, Mineka S, . A longitudinal examination of stress generation in depressive and anxiety disorders. J Abnorm Psychol. 2012;121(1):4-15. doi:10.1037/a0025835 22004114

[45] Bullmore E. The Inflamed Mind: A Radical New Approach to Depression. Picador; 2018.

[46] Franceschi C, Campisi J. Chronic inflammation (inflammaging) and its potential contribution to age-associated diseases. J Gerontol A Biol Sci Med Sci. 2014;69(suppl 1):S4-S9. doi:10.1093/gerona/glu057 24833586

[47] Ploubidis GB, Batty GD, Patalay P, Bann D, Goodman A. Association of early-life mental health with biomarkers in midlife and premature mortality. JAMA Psychiatry. 2020;78(1):38-46. doi:10.1001/jamapsychiatry.2020.2893 32997099 PMC7527946

[48] Studenski S, Perera S, Patel K, . Gait speed and survival in older adults. JAMA. 2011;305(1):50-58. doi:10.1001/jama.2010.1923 21205966 PMC3080184

