# Supplementary material for: Association of History of Psychopathology With Accelerated Aging at Midlife
Source: JAMA Psychiatry. 2021 Feb 17;78(5):1–10. doi: 10.1001/jamapsychiatry.2020.4626 (PMC7890535; doi:10.1001/jamapsychiatry.2020.4626)
Supplement: Supplement. — eAppendix 1. Measuring Psychopathology eAppendix 2. Modeling the Structure of Psychopathology eAppendix 3. Measuring Accelerated Aging eAppendix 4. Measuring Childhood Maltreatment and SES eAppendix 5. Attrition Analysis eTable 1. Standardized Factor Loadings for Models of the Structure of Psychopathology eTable 2. Correlations Between Laboratory/Clinical Test Measures and Self-Report Measures of Signs of Aging at Age 45 eTable 3. Association Between Childhood Health and Adult Psychopathology and Signs of Accelerated Aging eTable 4. Sensitivity Analyses of Associations Between P and Signs of Aging eFigure 1. Structure of Mental Disorder Data Collected in the Dunedin Study eFigure 2. Confirmatory Factor Analysis Models of the Structure of Psychopathology eFigure 3. Plot of the Positive Correlation Between the Variety and Persistence of Mental Disorders and P-Factor Scores in the Dunedin Study eReferences [file jamapsychiatry-e204626-s001.pdf]

Supplementary Online Content

Wertz J, Caspi A, Ambler A, et al. Association of history of psychopathology with accelerated aging at midlife. *JAMA Psychiatry*. Published online February 17, 2021. doi:10.1001/jamapsychiatry.2020.4626

eAppendix 1. Measuring Psychopathology
eAppendix 2. Modeling the Structure of Psychopathology
eAppendix 3. Measuring Accelerated Aging
eAppendix 4. Measuring Childhood Maltreatment and SES
eAppendix 5. Attrition Analysis
eTable 1. Standardized Factor Loadings for Models of the Structure of Psychopathology
eTable 2. Correlations Between Laboratory/Clinical Test Measures and Self-Report Measures of Signs of Aging at Age 45
eTable 3. Association Between Childhood Health and Adult Psychopathology and Signs of Accelerated Aging
eTable 4. Sensitivity Analyses of Associations Between P and Signs of Aging
eFigure 1. Structure of Mental Disorder Data Collected in the Dunedin Study
eFigure 2. Confirmatory Factor Analysis Models of the Structure of Psychopathology
eFigure 3. Plot of the Positive Correlation Between the Variety and Persistence of Mental Disorders and P-Factor Scores in the Dunedin Study
eReferences

This supplementary material has been provided by the authors to give readers additional information about their work.

© 2021 Wertz J et al. *JAMA Psychiatry*.

eAppendix 1: Measuring psychopathology

Mental disorders are disturbances in thought, behavior, and emotion that interfere with or limit social, family, educational, or work activities. In the Dunedin Study, these were identified according to the criteria of the Diagnostic and Statistical Manual of Mental Disorders (DSM) as previously described.[1] Psychiatric interviews were carried out by health professionals, not lay interviewers. At ages 18, 21, 26, 32, 38, and 45, interviews were carried out with the Diagnostic Interview Schedule.[2,3] The following disorders were assessed: Externalizing disorders (Attention-Deficit/Hyperactivity Disorder, Conduct Disorder, Alcohol Dependence, Tobacco Dependence, Cannabis Dependence, Other Drug Dependence), Internalizing disorders (Generalized Anxiety Disorder, Depression, Fears [including Social Phobia, Simple Phobia, Agoraphobia, Panic Disorder], Eating Disorders [including Bulimia and Anorexia], Posttraumatic Stress Disorder), and Thought disorders (Obsessive-Compulsive Disorder, Mania, Schizophrenia) (**eFigure 1**). To allow the study of comorbidity, multiple diagnoses could be assigned to a study member at once. However, DSM exclusionary criteria were applied (e.g., hallucinations better explained by drug use were not counted toward schizophrenia; generalized anxiety disorder was not diagnosed if the anxiety stemmed solely from fear about public speaking). The diagnoses were made using computerized algorithms matching the DSM criteria, and additionally requiring self-reported impairment ratings. For disorders where self-reports can be compromised by lack of insight (such as schizophrenia, mania), we also turned to information from additional sources, such as interviews with parents, systematic questionnaires mailed to informants who know the study member well (present data for 97% of the cohort), standardized clinical staff ratings (of observed behavior, such as poor grooming or bizarre speech, during the day of assessment, completed by interviewers immediately after interviews), and electronic medical records for each cohort member from the New Zealand national health system. In the case of schizophrenia and mania, narrative dossiers of symptoms were reviewed by experienced psychiatrists to achieve diagnostic consensus. These details are reported in our previous publications.[4]

At ages 18 and 21, diagnoses were made according to DSM-III-R;[5] at ages 26, 32, and 38, according to DSM-IV;[6] at age 45 according to the now-current DSM-V[7] (with the exception of substance-dependence disorders which were diagnosed according to DSM-IV, given that DSM-V dropped the distinction between abuse and dependence).

eAppendix 2: Modelling the structure of psychopathology

To evaluate the structure of psychopathology we used data from 6 adult assessments, carried out at ages 18, 21, 26, 32, 38, and 45 years, as previously described.[1] We studied DSM-defined symptoms of the following disorders that were repeatedly assessed in our longitudinal study: ADHD, Conduct Disorder, Alcohol Dependence, Cannabis Dependence, Dependence on Hard Drugs, Tobacco Dependence (assessed with the Fagerström Test for Nicotine Dependence[8]), Depression, Generalized Anxiety Disorder, Fears/Phobias (Social Phobia, Simple Phobia, Agoraphobia, Panic Disorder), PTSD, Eating Disorders (Anorexia, Bulimia), Obsessive-Compulsive Disorder, Mania, and positive and negative Schizophrenia symptoms. Ordinal measures represented the number of the observed DSM-defined symptoms associated with each disorder. Fears/phobias were assessed as the count of diagnoses for simple phobia, social phobia, agoraphobia, and panic disorder that a study member reported at each assessment. Of the 14

© 2021 Wertz J et al. *JAMA Psychiatry*.

disorders, 6 were not assessed at every occasion, but each disorder was measured at least three times (**eFigure 1**). Of the original 1,037 participants, we included 1,000 participants who had symptom count assessments for at least one age (845 participants had present symptom counts for all six assessments, 90 for five, 30 for four, 13 for three, and 14 for two). The 37 excluded participants comprised those who died (N=13) or left the Study (N=21) before age 18 or who had such severe developmental disabilities (N=3) that they could not be interviewed with the Diagnostic Interview Schedule.

Using Confirmatory Factor Analysis (CFA), we previously[9] tested two standard models that are frequently used to examine the structure of psychopathology:[10] (a) a correlated-factors model and (b) a hierarchical or bifactor model. In CFA, latent continuous factors are hypothesized to account for the pattern of covariance among observed variables. Our CFAs were run as multitrait-multimethod models. In these models, observed variables represented each of the disorders with a symptom scale at each assessment age (e.g., alcohol dependence was measured with a symptom scale at ages 18, 21, 26, 32, 38, and 45). Each model also included method/state factors designed to pull out age- and assessment-related variance (e.g., interviewer effects, mood effects, and age-specific vulnerabilities) that was uncorrelated with trait propensity toward psychopathology. Because symptom-level data are ordinal and have highly skewed distributions, we used polychoric correlations when testing our models. Polychoric correlations provide estimates of the Pearson correlation by mapping thresholds to underlying normally distributed continuous latent variables that are assumed to give rise to the observed ordinal variables. All CFA analyses were performed in Mplus version 8.3[11] using the weighted least squares means and variance adjusted (WLSMV) algorithm.[12] We assessed how well each model fit the data using the chi-square value, the comparative fit index (CFI), the Tucker-Lewis index (TLI), and the root-mean-square error of approximation (RMSEA). CFI values greater than .95 and TLI values greater than .95 indicate good fit; RMSEA scores less than .05 are considered good.[13]

The correlated-factors model (**eFigure 2, Model A**) tests the hypothesis that there are latent trait factors, each of which influences a subset of the diagnostic symptoms. We tested three factors representing Externalizing (with loadings from ADHD, conduct disorder, alcohol, cannabis, tobacco, and other drug dependence), Internalizing (with loadings from MDE, GAD, fears/phobias, PTSD, and eating disorders), and Thought Disorder (with loadings from OCD, mania, and schizophrenia). The model fit the data well: χ^2(2465, N=1,000) = 4082.230, CFI = .933, TLI = .929, RMSEA = .026, 90% confidence interval (CI) = [.024, .027]. As described previously[1] and shown in **eTable 1**, loadings on the three specific factors were all positive, generally high (all ps < .001), and averaged .790—Externalizing: average loading = .743; Internalizing: average loading = .814; Thought Disorder: average loading = .844. Correlations between the three factors were all positive and ranged from .420 between Internalizing and Externalizing to .847 between Internalizing and Thought Disorder. Thus, this model confirmed that three correlated factors (i.e., Internalizing, Externalizing, and Thought Disorder) explain well the structure of the disorder symptoms examined across 27 years of adulthood.

The hierarchical or bifactor model (**eFigure 2, Model B**) tests the hypothesis that the symptom measures reflect both General Psychopathology and three narrower styles of psychopathology. General Psychopathology (labeled *p* in **eFigure 2, Model B**) is represented by a factor that directly influences all of the diagnostic symptom factors. In addition, styles of psychopathology are represented by three factors, each of which influences a smaller subset of the symptom items. For example, alcohol symptoms load jointly on the General Psychopathology factor and on the

Externalizing style factor. The specific factors represent the constructs of Externalizing, Internalizing, and Thought Disorder over and above General Psychopathology. Model B had a Heywood case, an estimated variance that was negative for one of the lower-order disorder/symptom factors (specifically, mania), suggesting this was not a valid model. Inspection of the results revealed the source of the convergence problem. Specifically, the Thought Disorder factor was subsumed in *p*; that is, in the hierarchical model, symptoms of OCD, mania, and schizophrenia loaded very highly on *p*, but unlike symptoms of Externalizing and Internalizing, they could not form a separate Thought Disorder factor independently of *p*. We respecified the model accordingly, depicted in **Model B′ eFigure 2**. This model fit the data well: χ^2(2457, N=1,000) = 3695.364, CFI = .949, TLI = .945, RMSEA = .022, 90% CI [.021, .024]. As described previously [1] and shown in **eTable 1**, loadings on the General factor (*p*) were all positive, generally high (all ps < .001), and averaged .612; the highest standardized loadings were for mania (.976), schizophrenia (.865), PTSD (.860), and OCD (.772). **eFigure 3** shows that the *p* factor captures how cohort members differ from each other in the variety and persistence of many different kinds of disorders over the adult life course. Cohort members with higher *p* scores experienced a greater number of psychiatric disorders from adolescence to midlife (r=.76 [95% CI: .74, .79]).

eAppendix 3: Measuring accelerated aging

<u>Measuring Pace of Aging</u>

Age 26-45 lab test of Pace of Aging

Our measure of the Pace of Aging is detailed elsewhere.[14,15] Our approach is guided by geroscience theory's specification that measured aging has 3 key features that distinguish it from illnesses: (1) physiological decline in one direction, (2) continuing over years of time, (3) simultaneously involving all organ systems. We operationalized this theory by modelling (1) growth curves of decline in one direction, (2) over 20 years in 4 waves of biomarker data, and (3) using biomarkers that tap the function of multiple different organ systems.

We measured longitudinal changes in 19 biomarkers at ages 26, 32, 38 and 45 assessing cardiovascular, metabolic, pulmonary, renal, immune, and dental systems, totalling 69,715 data points (participants x biomarkers x assessments): Body mass index (BMI), Waist-hip ratio, Glycated hemoglobin, Leptin, Blood pressure (mean arterial pressure), Cardiorespiratory fitness (VO_2Max), Total cholesterol, Triglycerides, High-density lipoprotein (HDL), Lipoprotein(a), Apolipoprotein B100/A1 ratio, Forced vital capacity ratio (FEV_1/FVC), Forced expiratory volume in one second (FEV_1), estimated Glomerular Filtration Rate (eGFR), Blood Urea Nitrogen (BUN), high-sensitivity C-reactive Protein (hs-CRP), White blood cell count, mean periodontal attachment loss (AL), and dental-caries-affected tooth surfaces.

We then calculated each Study member's Pace of Aging in three steps. In the first step, we transformed the biomarker values to a standardized scale. For each biomarker at each wave, we standardized values according to the age-26 distribution (i.e. set to mean of 0 and a standard deviation of 1). Standardization was conducted separately for men and women. Standardized biomarker values greater than zero indicated levels that were "older" and values less than zero indicated levels "younger" than the average 26-year-old. To match, scores were reversed for VO_2Max, FEV_1/FVC, FEV_1, eGFR, and HDL cholesterol, which are known to decline with age.

Over the 2 decades of follow-up, the biomarkers in the panel indicated a progressive deterioration of physiological integrity with advancing chronological age; i.e. their cohort mean values tended to increase (i.e., worsen) from the age-26 assessment to the age-45 assessment.

In the second step, we calculated each Study member's slope for each of the 19 biomarkers—the average year-on-year change observed over the 2-decade period. Slopes were estimated using a mixed-effects growth model that regressed the biomarker's level on age. For only four of the 19 biomarkers we examined, cohort mean levels did not worsen over time as expected based on published associations with age-related chronic disease: white blood cell count and CRP levels remained stable with age; HDL cholesterol and apolipoprotein B100/A1 ratio improved with age. However, individual-difference slopes for these biomarkers did show the expected pattern of correlation with other biomarkers' slopes. For example, Study members whose apolipoprotein B100/A1 ratio increased during the follow-up period also showed increasing adiposity, declining lung function, and increasing systemic inflammation. We retained all pre-registered biomarkers in the Pace of Aging model.

In the third step, we combined information from the 19 slopes of the biomarkers to calculate each Study member's personal "Pace of Aging." Because we did not have any *a priori* basis for weighting differential contributions of the biomarkers to an overall Pace of Aging measure, we combined information using a unit-weighting scheme (all biomarkers were standardized to have mean=0, SD=1 based on their age-26 distributions, so slopes were denominated in comparable units). We calculated each Study member's Pace of Aging as the sum of age-dependent annual changes in biomarker Z-scores. Because the Dunedin birth cohort represents its population, its mean and distribution represent population norms. We used these norms to scale the Pace of Aging to reflect physiological change relative to the passage of time. We set the cohort mean Pace of Aging as a reference value equivalent to the physiological change expected during a single chronological year. Using this reference value, we rescaled Pace of Aging in terms of years of physiological change per chronological year (M = 1, SD = 0.29). On this scale, cohort members ranged in their Pace of Aging from 0.4 years of physiological change per chronological year (slow) to 2.4 years of physiological change per chronological year (fast).

Age-45 self-reported perceived age

We analyzed self-reported perceived age, because studies have linked subjective age with disease burden and cognitive functioning in old age, as well as mortality.[16–18] Self-perceived age at age 45 was measured using participants' response to the question "Many people feel younger or older than they really are. What age do you feel most of the time?".[17,19] The mean self-perceived age in 45-year old participants was M=40 (SD=8), with a range of 18-98 years. For our analyses we used the full range of the variable, but conducted sensitivity analyses in which we excluded n=27 people who rated their age as younger than 25 or older than 65. This did not change the pattern of results.

Childhood measure of poor physical health

We measured childhood health from medical exams, anthropometry, lung function testing, and clinical interviews with parents at assessments spanning birth to age 11 years as previously described (but without including motor development).[20,21] Children's overall health at birth, ages 3, 5, 7, 9, and 11 years was rated by two Unit staff members based on review of birth records and assessment dossiers including clinical assessments and reports of infections,

© 2021 Wertz J et al. *JAMA Psychiatry*.

diseases, injuries, hospitalizations, and other health problems collected from children's mothers during standardized interviews. Ratings were made on a five-point scale (inter-rater agreement=0.85) and reverse-coded before constructing summary measure. Body mass index was calculated from height and weight measurements taken at ages 5, 7, 9, and 11 years. In addition, tricep and subscapular skinfold thicknesses were measured at ages 7 and 9 years by trained anthropometrists.[22] (For calculation of the overall measure, tricep and subscapular skinfold thicknesses were averaged to create a single score.) Systolic and diastolic blood pressure were measured at ages 7, 9, and 11 years using a London School of Hygiene and Tropical Medicine blind mercury sphygmomanometer (Cinetronics Ltd., Mildenhall, United Kingdom).[23] Fixed expiratory volume in one second (FEV1) and the ratio of FEV1 to forced vital capacity (FVC) were measured at ages 9 and 11 using a Godart water-sealed spirometer[24] and reverse-coded before constructing summary measures. To construct a cross-age measure of childhood physical health, assessments were standardized to M=0 SD=1 within age and sex and then averaged across ages. The measure was reverse-coded so that higher scores indicated poorer childhood physical health.

Measuring social hearing difficulties

Age-45 lab test of social hearing

Social hearing refers to listening ability in noisy environments, which requires complex processing ability. We analyzed social hearing because the ability to recognize speech in noisy environments declines with age, and deterioration in social hearing has been linked with cognitive decline.[25,26] To measure social hearing, participants completed the Listening in Spatialised Noise–Sentences Test (LiSN-S) (Phonak, Switzerland). All hearing tests were carried out in an acoustically attenuated room with a double door and sound-absorbing wall covering while wearing headphones. Auditory stimuli were delivered through a pair of Sennheiser 215 headphones attached to a Mini PCM2704 external sound card configured by Phonak. The LiSN-S produces a three-dimensional auditory environment through the headphones via four different task conditions.[27] Target sentences spoken by a female speaker are superimposed with distractor stories (maskers). Across the four conditions, these maskers differ with respect to perceived spatial location (0° or $\pm 90^{o}$ azimuth), and speaker identity (same or different to the target speaker). The following order of conditions was identically presented to all participants: 1) different speaker at $\pm 90^{o}$ azimuth, 2) same speaker at $\pm 90^{o}$ azimuth, 3) different speaker at $\pm 0^{o}$ azimuth, and 4) same speaker at $\pm 0^{o}$ azimuth.

The masking stories were consistently presented at an intensity of 55 decibels sound pressure level (dB SPL). Participants repeated the target sentences and were scored in the software on their accuracy (words correct in each sentence). The program was adaptive, with target sentences delivered at 62 dB SPL to start, and intensity levels continuously adjusted up (if <50% of the words in the sentence correct), and down (if >50% of the words in the sentence correct), based on accuracy. The first few sentences (a minimum of 5) are considered practice sentences. This practice testing continues where levels were lowered in 4 dB increments, until one upward reversal in performance was recorded (i.e. the sentence score drops <50% of words correct), after which the increments decreased to ±2 dB steps. Up to 30 sentences are presented in each of four conditions. Testing stops in a particular condition when the listener has either (a) completed the entire 30 sentences in any one condition; or (b) completed the practice sentences plus a minimum of a further 17 scored sentences, and their standard error, calculated

© 2021 Wertz J et al. *JAMA Psychiatry*.

automatically in real time over the scored sentences, is less than 1 dB. The test condition continued until the average of the levels from positive-and negative-going reversals amounted to ≥3 (independent midpoint target level), and the standard error of these midpoints was less than 1 dB. Alternatively, the test condition continued until it reached the maximum number of 30 sentence presentations. Speech-reception thresholds were calculated as the lowest intensity at which the individual could repeat 50% of the words correctly. Our primary outcome measure was the speech-reception threshold from the low-cue condition, representing performance in the most difficult auditory environment (masker speaker same as the target speaker, and masker was presented at 0° azimuth, in the same location as the target speaker). This reflects hearing when the person is not receiving optimum auditory information. A higher score indicates worse performance.

Impairments in social hearing may be due to peripheral hearing decline (i.e. the detection of the auditory signal) rather than declines in social hearing per se. To test this possibility, we re-ran our analysis of associations between *p* and social hearing whilst controlling for hearing ability averaged across four frequencies (500 Hz, 1000 Hz, 2000 Hz, and 4000 Hz), assessed using pure-tone audiometry at age 45. Controlling for hearing ability (in addition to sex) did not significantly reduce associations between *p* and social hearing (from β=.18 [95%CI .12, .24] p<.01 before adjustment) to β=.16 [95%CI .10, .22], p<.01 after adjustment).

Age-45 self-report of hearing difficulties

Participants completed a hearing questionnaire, including three questions from the 12-item Speech, Spatial, and Qualities of Hearing Scale (SSQ12)[28] about whether they could 1) follow a conversation in a group in a busy restaurant, 2) follow a conversation in a group where the conversation switches between speakers, and 3) tell immediately where a dog is by hearing its bark. Participants responded on a scale of 0 (not at all) to 10 (perfectly). A total score was constructed by summing across items (M=7.84, SD=5.15, Range 0-29).

Childhood measure of social hearing difficulties

At age 11, a speech-in-noise (SPIN) test was administered using a tape supplied by the Audiology Centre in Auckland. Six Arthur Boothroyd word lists with 10 words each[29] were presented in the following order: (1) List 1 (no noise) (2) List 2 (10 db signal/noise ratio), (3) List 3 (5 db signal/noise ratio), (4) List 4 (5 db signal/noise ratio), (5) List 5 (10 db signal/noise ratio), (6) List 6 (no noise).[30] Words were spoken by a New Zealand male and presented at 60 dbSPL. The tape was played on a Technics stereo cassette deck model M215 attenuated through an Interacoustics AC3 Clinical Audiometer calibrated to ISO (1064) standards. Children's responses were scored phonemically as follows: 3 for a single phoneme; 7 for 2 phonemes; and 10 for the whole word giving a maximum possible score for each list of 100. A summary score was constructed for each of the three conditions (no noise, 10 dB and 5 dB signal to noise ratio), reflecting the percentage of words correctly identified (no noise condition: mean M=98.4, SD=2.4, Range 68.5-100; 10 dB condition M=90.2, SD=4.6, Range 49.5-98.5; 5db condition: M=79.3, SD=7.0, Range 29-93.5). To control for childhood hearing when testing associations with our age-45 outcome, i.e. social hearing under the most difficult auditory environment, we used the score derived from the 5 dB signal-to-noise ratio condition, measuring hearing under the most difficult auditory environment. We reverse-coded the score to indicate greater difficulty with social hearing.

© 2021 Wertz J et al. *JAMA Psychiatry*.

Measuring vision difficulties

Age-45 lab tests of vision difficulties

We analyzed visual contrast sensitivity, because contrast sensitivity declines with age, even after adjusting for visual acuity;[31] can be more disabling than visual acuity loss,[32] and is a better predictor of mobility performance than visual acuity.[33] The ability to detect objects of different sizes at lower contrasts is expressed as a contrast sensitivity function (CSF) and determines the person's contrast detection threshold, the lowest contrast at which a pattern can be seen. Contrast sensitivity scores are linear on a logarithmic scale, and lower log CS values reflect worse contrast sensitivity. CSF testing was administered by trained visual technicians. Participants wore their glasses or contact lenses (if these were normally worn). Participants were seated one meter from the Thomson Test Chart and the Samsung 23" LCD Thin Client screen. Room lighting was set at 520 lux. Contrast sensitivity was tested with both eyes open. The Pelli-Robson chart presents three letters per line and the black letters gradually fade from black to grey to white on the white background to determine the lowest level of "contrast" that the eye can detect. If only one letter on a line was correctly determined by the study member, the number of letters was recorded to determine the CSF score. However, if two letters on a line were correctly determined, the technician proceeded to the next line to determine if the study member could correctly determine any of these letters. We reverse-coded the CFS score so that higher scores indicated lower contrast sensitivity.

Age-45 self-report of vision difficulties

Participants completed the 10-item Vision Quality of Life Core Measure (VCM1)[34] about vision-related quality of life, including items referring to physical, social and psychological issues (embarrassment, anger, depression, loneliness, fear of deterioration in vision, safety at home, safety outside the home, coping with everyday life, inability to do preferred activities and life interference) (example items "How often has your eyesight stopped you from doing the things you wanted to do?"; "How often has your eyesight made you concerned or worried about coping with everyday life?"; "How much has your eyesight interfered with your life in general?"). Participants responded on a scale of 0 (not at all) to 5 (all the time). A total score was constructed by summing across items (M=3.59, SD=4.72, Range 0-42).

Childhood measure of vision difficulties

At ages 7, 9 and 11 years, participants' visual acuity was assessed using standard testing as previously described.[35] At age 7 years, visual acuity was assessed using the Sheridan Gardiner single optotype letter matching test at 6m. At age 9, and 11 years, visual acuity was measured using a 4-m logarithmic test chart. Each eye was tested separately, and the contralateral eye was occluded. The tests were performed without glasses and repeated with glasses, if they were available. A pinhole was used if the visual acuity was 6/9 or worse and glasses were not available. Testing was done in the same well-lit room at each age. Acuity testing results were converted to logMAR scores so that measures across childhood were on the same scale. On the logMAR scale, a score of 1.0 is poor vision (6/60 or 20/200 on a usual chart), a score of 0 is good vision (6/6 or 20/20 on a usual chart), and a negative score is better than 6/6 vision. For each age, we constructed a 'best-eye' visual acuity score for each study member by assigning participants the highest score they had obtained at that age. To construct a cross-age measure of childhood visual acuity assessments were standardized to M=0 SD=1 within age and then

averaged across ages. The measure was reverse-coded so that higher scores indicated lower acuity.

Measuring balance difficulties

Age-45 lab test of balance difficulties

We analyzed balance, because difficulties with balance increase with age[36] and are associated with reduced mobility and risk for falls.[37] Balance was measured using the Unipedal Stance Test as the maximum time achieved across three trials of the test with eyes closed.[36,38,39] The measure was reverse-coded to indicate more balance difficulties.

Age-45 self-report of balance difficulties

Participants responded to four items about difficulties with balance (example item: "Do you have feelings that things are spinning or moving around?). Participants responded on a scale of 0 (no) ,1 (sometimes) and 2 (yes). A total score was constructed by summing across items (M=.76, SD=1.28, Range 0-8).

Childhood measure of balance difficulties

Balance was assessed at ages 3, 7 and 9 using the balance subtests of the Bayley Motor Scales (age 3) [40] and of the Basic Motor Ability Test (ages 7 and 9).[41] To construct a cross-age measure of childhood balance, assessments were standardized to M=0 SD=1 within age and then averaged across ages. The measure was reverse-coded so that higher scores indicated more balance difficulties.

Measuring motor difficulties

Age-45 lab test of motor difficulties

We analyzed gait speed because it is considered a geriatric vital sign and predicts multiple adverse outcomes, including frailty, disability, and mortality in older adults.[42] Gait speed (meters per second) was assessed with the 6-m-long GAITRite Electronic Walkway (CIR Systems, Inc) with 2-m acceleration and 2-m deceleration before and after the walkway, respectively, as previously described.[15] Gait speed was assessed under 3 walking conditions: usual gait speed (walk at normal pace from a standing start, measured as a mean of 2 walks) and 2 challenge paradigms, dual task gait speed (walk at normal pace while reciting alternate letters of the alphabet out loud, starting with the letter "A," measured as a mean of 2 walks) and maximum gait speed (walk as fast as safely possible, measured as a mean of 3 walks). Gait speed was correlated across the 3 walk conditions.[15] To increase reliability and take advantage of the variation in all 3 walk conditions (usual gait and the 2 challenge paradigms), we calculated the mean of the 3 highly correlated individual walk conditions to generate our primary measure of composite gait speed. We reverse-coded the measure so that a higher score indicates a slower gait-speed.

Age-45 self-report of motor difficulties

Participants completed the 10-item RAND 36-Item Health Survey 1.0 physical functioning scale,[43] about their difficulty with completing various activities, e.g., climbing several flights of stairs, walking more than 1 km, participating in strenuous sports, etc.

© 2021 Wertz J et al. *JAMA Psychiatry*.

Participants responded on a scale of 1 (limited a lot), 2 (limited a little) and 3 (not limited at all). A total score was constructed using RAND scoring instructions (https://www.rand.org/health-care/surveys_tools/mos/36-item-short-form/scoring.html). Scores were reversed to reflect physical limitations so that a high score indicates more limitations (M=10.50, SD=16.44, Range 0-100).

Childhood measure of motor difficulties

Motor development was assessed at age 3 years using the Bayley Motor Scales,[40] at age 5 years using the McCarthy Motor Scales,[44] and at ages 7 and 9 years using the Basic Motor Ability Test.[41] To construct a cross-age measure of childhood motor development, assessments were standardized to M=0 SD=1 within age, and then averaged across ages. The measure was reverse-coded so that higher scores indicated more motor difficulties.

<u>Measuring cognitive difficulties</u>

Age-45 lab test of cognitive difficulties

We analyzed cognitive functioning because cognitive ability declines with age[45] and predicts survival and health in old age.[46,47] Cognitive functioning was measured by administering the Wechsler Adult Intelligence Scale-IV (WAIS-IV)[48] to the participants at age 45 years, yielding a measure of full-scale IQ, standardized to M=100, SD=15. We reverse-coded the measure so that a higher score indicates more cognitive difficulties.

Age-45 self-report of cognitive difficulties

Participants completed a 22-item Cognitive Complaints interview about memory difficulties in the past year, including DSM-5 symptoms of mild neurocognitive disorder[7] and items from the Cognitive Failures Questionnaire (CFQ)[49] (e.g. “I have difficulty finding the word I want to use.”; “I have to make lists to remember to do things.”). This interview looks at cognitive impairment, which is a term used to describe any of a group of cognitive disorders including mild cognitive impairment, age-related cognitive decline, vascular dementia, decreased long-term memory formation and neurodegenerative diseases such as Alzheimer's disease and Parkinson's disease. Items (e.g. “I have difficulty finding the word I want to use.”; “I have to make lists to remember to do things.”). Participants responded on a scale of 0 (no), 1 (sometimes) and 2 (often). A total score was constructed by summing across items (M=11.12, SD=6.76, Range 0-44).

Childhood measure of cognitive difficulties

Participants’ cognitive functioning was individually assessed at ages 7, 9, and 11 years using the Wechsler Intelligence Scale for Children–Revised,[50] yielding a measure of full-scale IQ, standardized to M=100, SD=15 at each age. To construct a cross-age measure of childhood cognitive function, assessments were averaged across ages and standardized to M=0 and SD=1. The measure was reverse-coded so that higher scores indicated more cognitive difficulties.

<u>Measuring facial age</u>

© 2021 Wertz J et al. *JAMA Psychiatry*.

Facial Age was based on ratings by an independent panel of eight raters of each participant's digital facial photograph. Facial Age was based on two measurements of perceived age. First, Age Range was assessed by an independent panel of four raters, who were presented with standardized (non-smiling) facial photographs of participants and were kept blind to their actual age. Raters used a Likert scale to categorize each participant into a 5-year age range (i.e., from 20-24 years old up to 70+ years old) (interrater reliability = 0.77). Scores for each participant were averaged across all raters. Second, Relative Age was assessed by a different panel of four raters, who were told that all photos were of people aged 45 years old. Raters then used a 7-item Likert scale to assign a "relative age" to each participant (1="young looking", 7="old looking") (interrater reliability = .79). The measure of perceived age at 45 years, Facial Age, was derived by standardizing and averaging Age Range and Relative Age scores.

© 2021 Wertz J et al. *JAMA Psychiatry*.

eAppendix 4: Measuring childhood maltreatment and SES

Childhood maltreatment

Childhood maltreatment[51] includes evidence of (1) maternal rejection assessed at age 3 years by observational ratings of mothers' interaction with the study children, (2) harsh discipline assessed at ages 7 and 9 years by parental report of disciplinary behaviours, (3) 2 or more changes in the child's primary caregiver, and (4) physical abuse and (5) sexual abuse reported by study members once they reached adulthood. For each child, our cumulative index counts the number of maltreatment indicators during the first decade of life; 64.2% of children experienced no maltreatment, 26.6% experienced 1 indicator of maltreatment ("probable" maltreatment), and 9.2% experienced 2 or more indicators of maltreatment ("definite" maltreatment).

Childhood SES

Childhood socioeconomic status of participants' families was measured using a 6-point scale that assessed parents' occupational statuses, defined based on average income and educational levels derived from the New Zealand Census.[52]

© 2021 Wertz J et al. *JAMA Psychiatry*.

eAppendix 5: Attrition analysis

We conducted an attrition analysis using childhood socioeconomic status (SES), childhood IQ, childhood physical health and *p*-factor scores from age 18 years, to determine whether participants in the Phase-45 data collection were representative of the original cohort.

No significant differences were found between the full cohort, those deceased, those alive or those seen at Phase 45 on childhood SES.

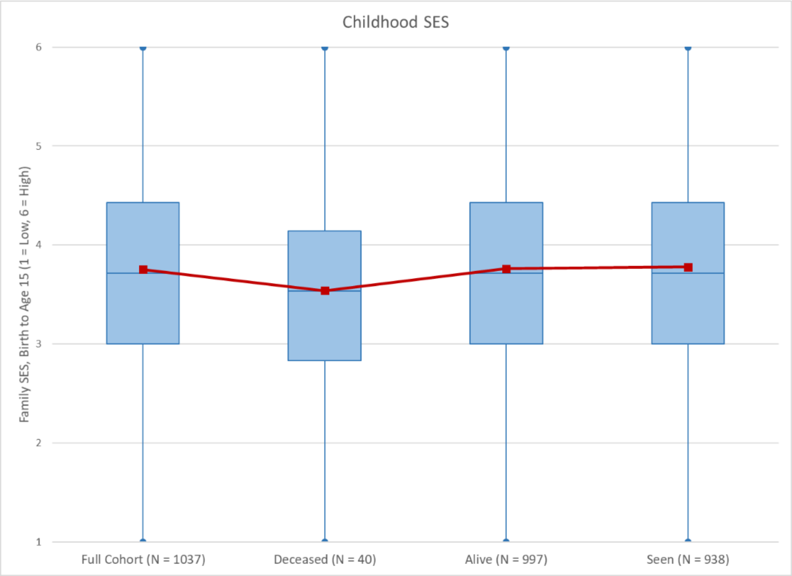

No significant differences in childhood IQ were found between the full cohort, those still alive, or those seen at Phase 45. Those who were deceased by the Phase 45 data collection had significantly lower childhood IQ's than those who were still alive (t=2.09, p=.04).

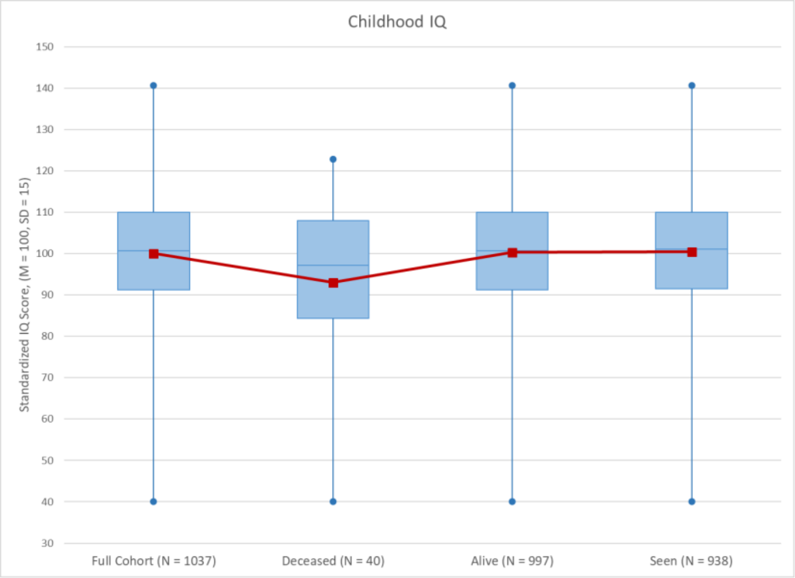

© 2021 Wertz J et al. *JAMA Psychiatry*.

No significant differences were found between the full cohort, those deceased, those alive or those seen at Phase 45 on childhood poor physical health.

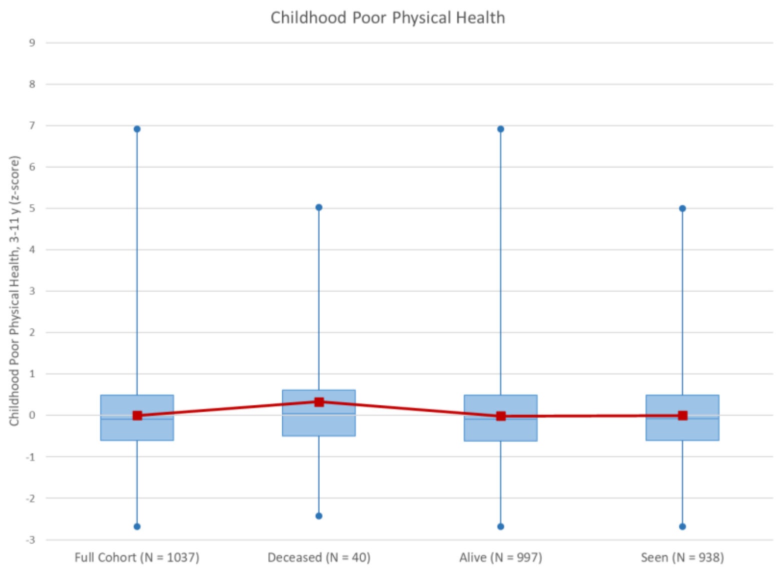

No significant differences in *p*-factor were found between the full cohort, those still alive, or those seen at Phase 45. Those who were deceased by the Phase 45 data collection had significantly higher p-factor scores than those who were still alive (t=-2.86, p=.004).

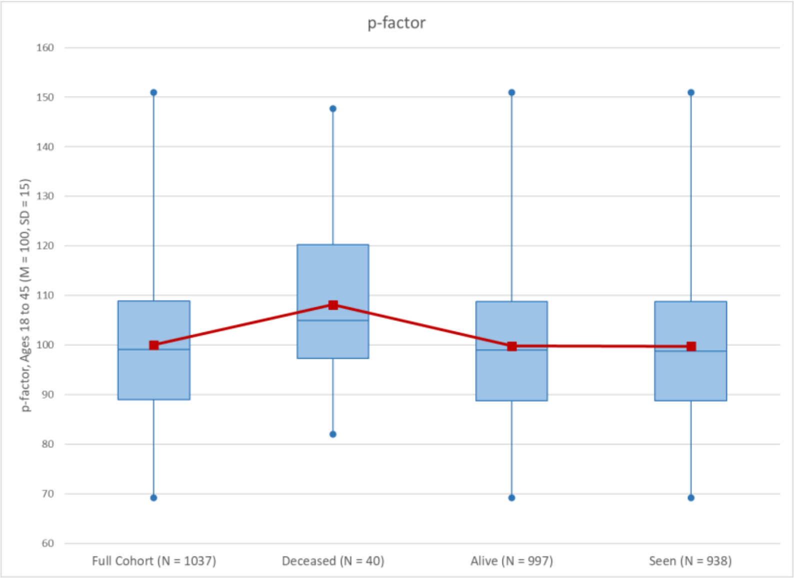

© 2021 Wertz J et al. *JAMA Psychiatry*.

eTable 1. Standardized Factor Loadings for Models of the Structure of Psychopathology

		Model A: Correlated Factors				Model B': Bifactor Model		
Model Fit Statistics								
	Chi-Square (WLSMV)	4082.230				3695.364		
	Degrees of Freedom	2465				2457		
	Comparative Fit Index	.933				.949		
	Tucker-Lewis Index	.929				.945		
	RMSEA [90% CI]	.026 [.024, .027]				.022 [.021, .024]		
		Externalizing	Internalizing	Thought		*p* factor	Externalizing	Internalizing
Standardized factor loadings								
	ADHD	.567				.595	.121	
	Alcohol	.651				.300	.622	
	Cannabis	.831				.369	.850	
	Hard drugs	.845				.466	.694	
	Tobacco	.675				.450	.468	
	Conduct disorder	.888				.504	.714	
	Major depression		.968			.768		.587
	Generalized anxiety		.892			.686		.642
	Fears/phobias		.717			.582		.424
	Eating disorder		.499			.377		.374
	PTSD		.994			.860		.351
	OCD			.739		.772		
	Mania			.955		.976		
	Schizophrenia			.838		.865		
Factor Correlations								
	Externalizing		.420	.622				
	Internalizing			.847				

© 2021 Wertz J et al. *JAMA Psychiatry*.

eTable 2. Correlations Between Laboratory/Clinical Test Measures and Self-Report Measures of Signs of Aging at Age 45

	Corresponding self-report
Lab test	r (95%CI)
Pace of Aging	.09 (.03, .16)
Hearing difficulties	.25 (.19, .31)
Vision difficulties	.20 (.14, .27)
Balance difficulties	.14 (.08, .21)
Motor difficulties	.30 (.23, .36)
Cognitive difficulties	.17 (.11, .24)

© 2021 Wertz J et al. *JAMA Psychiatry*.

eTable 3. Association Between Childhood Health and Adult Psychopathology and Signs of Accelerated Aging

	Psychopathology	Signs of accelerated aging	
Predictor	Outcome: *p*-factor score	Outcome: Lab test	Outcome: Self-report
	β (95%CI)	β (95%CI)	β (95%CI)
Childhood poor physical health	.11 (.05, .18)	.20 (.14, .27)[a]	.09 (.02, .16)[a]
Childhood poor hearing	.12 (.04, .19)	.12 (.04, .19)[b]	.14 (.07, .22)[b]
Childhood poor vision	.03 (-.04, .10)	.11 (.04, .18)[c]	.18 (.11, .25)[c]
Childhood poor balance	.02 (-.05, .09)	.15 (.08, .22)[d]	.05 (-.03, .12)[d]
Childhood poor motor skills	.11 (.04, .17)	.27 (.20, .33)[e]	.20 (.13, .27)[e]
Childhood poor cognitive function	.18 (.12, .25)	.78 (.75, .80)[f]	.18 (.11, .24)[f]

Table reports standardized estimates. All analyses are adjusted for participants' sex. CI: Confidence Interval.
[a] Adult outcome: Pace Of Aging.
[b] Adult outcome: Hearing difficulties.
[c] Adult outcome: Vision difficulties.
[d] Adult outcome: Balance difficulties.
[e] Adult outcome: Motor difficulties.
[f] Adult outcome: Cognitive difficulties.

© 2021 Wertz J et al. *JAMA Psychiatry*.

eTable 4. Sensitivity Analyses of Associations Between P and Signs of Aging

	Associations between *p* and aging				
	In all participants	Controlling for BMI	Controlling for smoking	Net of participants taking antipsychotic drugs	Net of participants with physical disease
	β (95%CI)[a]	β (95%CI)	β (95%CI)	β (95%CI)	β (95%CI)
Lab test					
Pace of Aging	.21 (.15, .27)	.20 (.16, .25)	.17 .11, .24)	.19 (.13, .25)	.22 (.15, .28)
Hearing difficulties	.16 (.09, .22)	.16 (.09, .22)	.14 (.08, .21)	.14 (.07, .20)	.15 (.08, .22)
Vision difficulties	.05 (-.02, .11)	.04 (-.02, .11)	.03 (-.04, .10)	.02 (-.05, .09)	.06 (-.01, .13)
Balance difficulties	.17 (.11, .23)	.16 (.10, .22)	.14 (.08, .21)	.15 (.09, .22)	.16 (.09, .22)
Motor difficulties	.14 (.08, .20)	.13 (.07, .19)	.14 (.07, .21)	.13 (.07, .20)	.14 (.08, .21)
Cognitive difficulties	.10 (.05, .14)	.10 (.05, .14)	.08 (.03, .12)	.08 (.04, .12)	.09 (.05, .14)
Self-report					
Pace of Aging	.07 (.00, .14)	.07 (.00, .14)	.08 (.01, .15)	.08 (.01, .14)	.08 (.01, .15)
Hearing difficulties	.20 (.14, .26)	.20 (.14, .26)	.20 (.13, .27)	.19 (.13, .25)	.20 (.13, .26)
Vision difficulties	.34 (.28, .39)	.33 (.28, .39)	.35 (.29, .41)	.32 (.26, .38)	.34 (.28, .40)
Balance difficulties	.32 (.26, .38)	.32 (.26, .38)	.35 (.29, .40)	.32 (.27, .38)	.32 (.26, .38)
Motor difficulties	.34 (.28, .40)	.33 (.27, .39)	.36 (.30, .42)	.33 (.27, .39)	.34 (.27, .40)
Cognitive difficulties	.44 (.38, .49)	.44 (.38, .49)	.47 (.42, .52)	.43 (.38, .49)	.44 (.38, .49)

Note: All models are adjusted for sex, childhood health, childhood maltreatment, and childhood SES. CI=Confidence Interval.

© 2021 Wertz J et al. *JAMA Psychiatry*.

eFigure 1.

Structure of Mental Disorder Data Collected in the Dunedin Study

	18y	21y	26y	32y	38y	45y
**EXTERNALIZING DISORDERS**						
ADHD	●				●	●
Conduct Disorder	●	●	●	●	●	●
Alcohol Dependence	●	●	●	●	●	●
Tobacco Dependence	●	●	●	●	●	●
Cannabis Dependence	●	●	●	●	●	●
Drug Dependence			●	●	●	●
**INTERNALIZING DISORDERS**						
Anxiety (GAD)	●	●	●	●	●	●
Depression (MDE)	●	●	●	●	●	●
Fears (any Panic, SIP, SOP, Agoraphobia)	●	●	●	●	●	●
Eating Disorder (any bulimia, anorexia)	●	●	●			
PTSD			●	●	●	●
**THOUGHT DISORDERS**						
Obsessive Compulsive Disorder	●	●	●	●	●	●
Mania		●	●	●	●	●
Schizophrenia		●	●	●	●	●

Note: The chart shows the age at which each disorder was assessed. Although each disorder was not assessed at every age, each disorder was assessed on at least three occasions.

© 2021 Wertz J et al. *JAMA Psychiatry*.

eFigure 2.

Confirmatory Factor Analysis Models of the Structure of Psychopathology

Model A

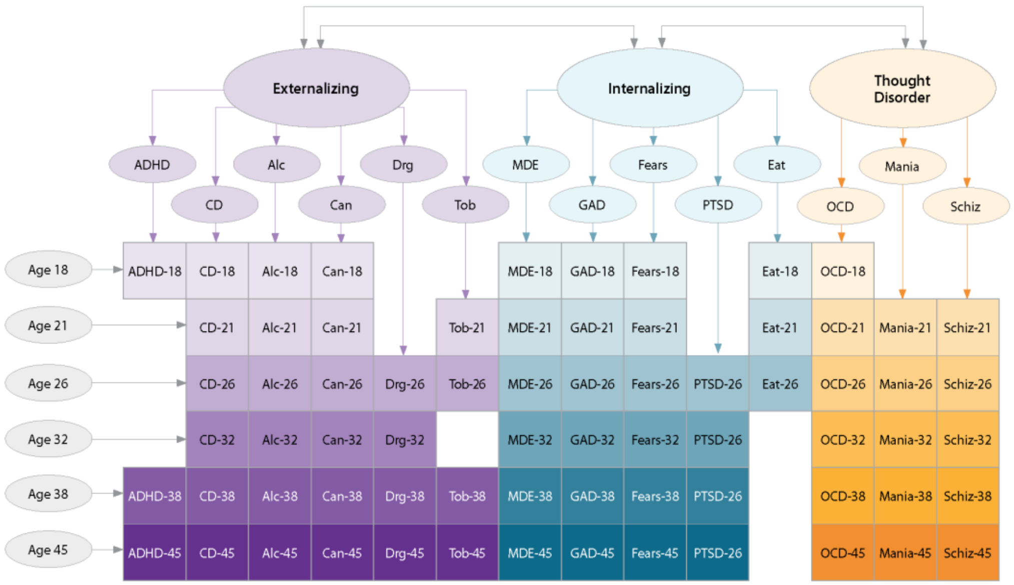

Note: **Model A** is the correlated-factors model. Using this model, we tested the hypothesis that there are latent trait factors, each of which influences a subset of the diagnostic symptoms. We tested three factors representing Externalizing (with loadings from ADHD, conduct disorder, alcohol dependence, cannabis dependence, drug dependence and tobacco dependence), Internalizing (with loadings from MDE, GAD, fears/phobias, PTSD, and eating disorders), and Thought Disorder (with loadings from OCD, mania, and schizophrenia).

© 2021 Wertz J et al. *JAMA Psychiatry*.

Model B

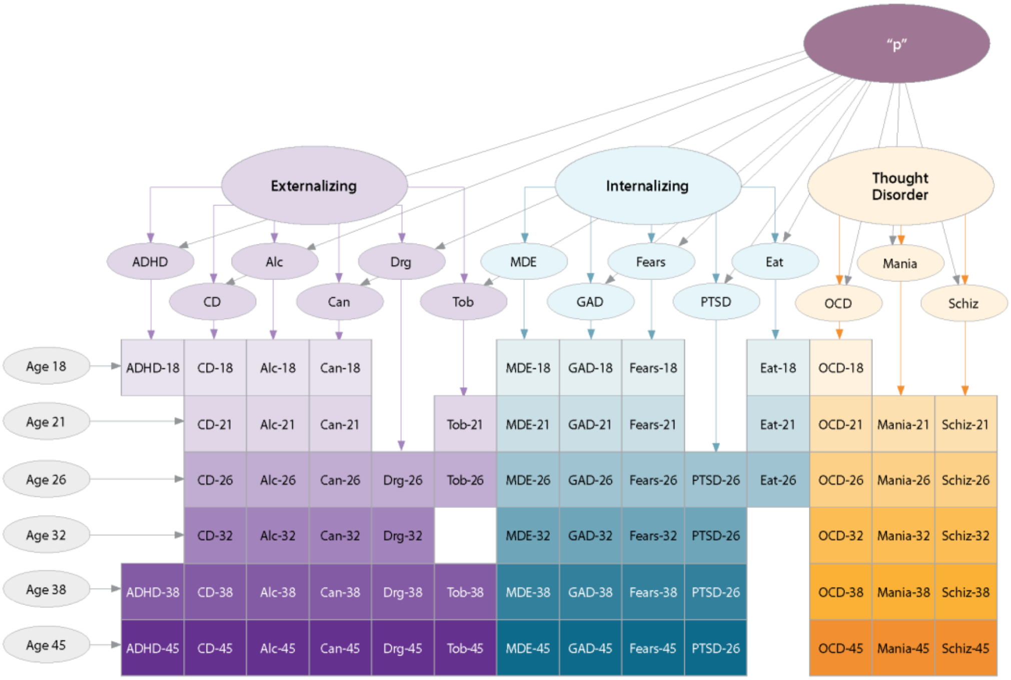

Note: **Model B** is the hierarchical or bifactor model. Using this model, we tested the hypothesis that the symptom measures reflect both General Psychopathology and three narrower styles of psychopathology. General Psychopathology (labeled *p* in Model B) is represented by a factor that directly influences all of the diagnostic symptom factors. In addition, styles of psychopathology are represented by three factors, each of which influences a smaller subset of the symptom items. For example, alcohol symptoms load jointly on the General Psychopathology factor and on the Externalizing style factor. The specific factors represent symptoms of Externalizing, Internalizing, and Thought Disorder that are independent of General Psychopathology.

© 2021 Wertz J et al. *JAMA Psychiatry*.

Model B’

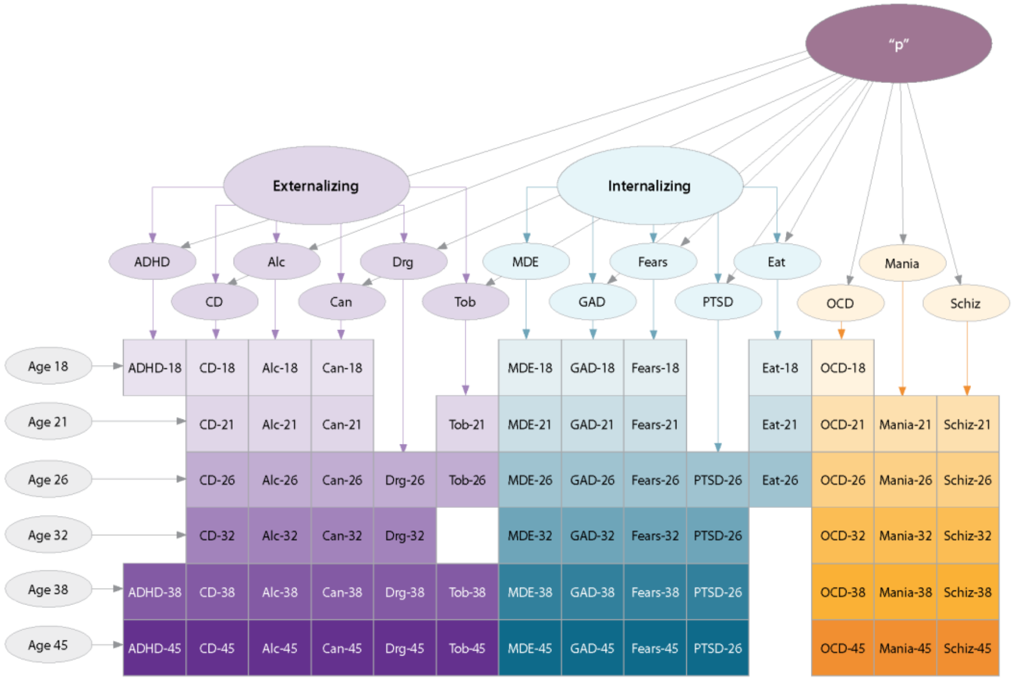

Note: Model B had a Heywood case, an estimated variance that was negative for one of the lower-order disorder/symptom factors (specifically, mania), suggesting this was not a valid model. Inspection of the results revealed the source of the convergence problem. Specifically, the Thought Disorder factor was subsumed in *p*; that is, in the hierarchical model, symptoms of OCD, mania, and schizophrenia loaded very highly on *p*, but unlike symptoms of Externalizing and Internalizing, they could not form a separate Thought Disorder factor independently of *p*. We respecified the model accordingly, depicted in **Model B′**.

© 2021 Wertz J et al. *JAMA Psychiatry*.

eFigure 3.

Plot of the Positive Correlation Between the Variety and Persistence of Mental Disorders and P-Factor Scores in the Dunedin Study

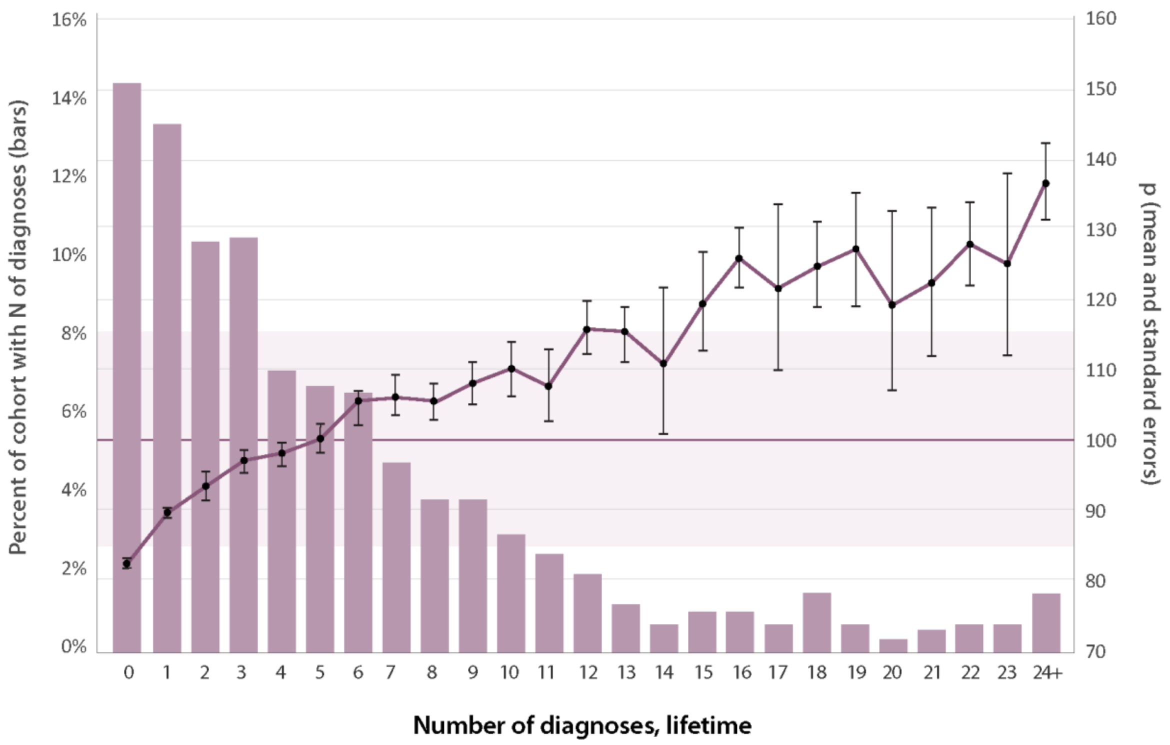

Note: The *p* factor captures how participants differ from each other in the variety and persistence of many different kinds of disorders over the life course. Participants with higher *p* scores experienced a greater number of mental disorders from adolescence to midlife (r=.76 [95% CI: .74, .79]). Shaded area represents ± 1 SD for *p*.

© 2021 Wertz J et al. *JAMA Psychiatry*.

eReferences

1. Caspi A, Houts RM, Ambler A, et al. Longitudinal assessment of mental health disorders and comorbidities across 4 decades among participants in the Dunedin birth cohort study. *JAMA Netw open*. 2020;3(4):e203221. doi:10.1001/jamanetworkopen.2020.3221
2. Robins LN, Cottler L, Bucholz KK, Compton W. *Diagnostic Interview Schedule for DSM-IV*. St Louis, MO: Washington University School of Medicine; 1995.
3. Robins LN, Helzer JE, Croughan J, Ratcliff KS. National Institute of Mental Health Diagnostic Interview Schedule: Its history, characteristics, and validity. *Arch Gen Psychiatry*. 1981;38(4):381-389. doi:10.1001/archpsyc.1981.01780290015001
4. Meier MH, Caspi A, Reichenberg A, et al. Neuropsychological decline in schizophrenia from the premorbid to the postonset period: Evidence from a population-representative longitudinal study. *Am J Psychiatry*. 2014;171(1):91-101. doi:10.1176/appi.ajp.2013.12111438
5. APA. *American Psychological Association: Diagnostic and Statistical Manual of Mental Disorders*. Revised Th. Washington, DC: American Psychiatric Association; 1987.
6. APA. *Diagnostic and Statistical Manual of Mental Disorders (4th Ed., Text Rev.)*. Washington, DC: American Psychiatric Association; 2000.
7. APA. *Diagnostic and Statistical Manual of Mental Disorders (5th Ed.)*. Washington, DC: American Psychiatric Association; 2013.
8. Heatherton TF, Kozlowski LT, Frecker RC, Fagerstrom K-OO. The Fagerström Test for Nicotine Dependence: A revision of the Fagerstrom Tolerance Questionnaire. *Br J Addict*. 1991;86(9):1119-1127. doi:10.1111/j.1360-0443.1991.tb01879.x
9. Caspi A, Houts RM, Belsky DW, et al. The p Factor: One General Psychopathology Factor in the Structure of Psychiatric Disorders? *Clin Psychol Sci*. 2013;2(2):119-137. doi:10.1177/2167702613497473
10. Caspi A, Moffitt TE. All for one and one for all: Mental disorders in one dimension. *Am J Psychiatry*. 2018;175(9):831-844. doi:10.1176/appi.ajp.2018.17121383
11. Muthén LK, Muthén BO. *Mplus User's Guide. Eighth Edition.* Los Angeles, CA: Muthén & Muthén
12. Asparouhov T, Muthen B. *Weighted Least Squares Estimation with Missing Data*.; 2010.
13. Bollen KA, Curran PJ. *Latent Curve Models: A Structural Equation Approach.* Hoboken, NJ: Wiley; 2006.
14. Belsky DW, Caspi A, Houts R, et al. Quantification of biological aging in young adults. *Proc Natl Acad Sci U S A*. 2015;112(30):E4104-E4110. doi:10.1073/pnas.1506264112
15. Rasmussen LJH, Caspi A, Ambler A, et al. Association of neurocognitive and physical function with gait speed in midlife. *JAMA Netw Open*. 2019;2(10):e1913123. doi:10.1001/jamanetworkopen.2019.13123
16. Stephan Y, Caudroit J, Jaconelli A, Terracciano A. Subjective age and cognitive functioning: A 10-year prospective study. *Am J Geriatr Psychiatry*. 2014;22(11):1180-1187. doi:10.1016/j.jagp.2013.03.007
17. Stephan Y, Sutin AR, Caudroit J, Terracciano A. Subjective age and changes in memory in older adults. *Journals Gerontol Ser B Psychol Sci Soc Sci*. 2016;71(4):675-683. doi:10.1093/geronb/gbv010
18. Kotter-Grühn D, Kleinspehn-Ammerlahn A, Gerstorf D, Smith J. Self-perceptions of aging predict mortality and change with approaching death: 16-year longitudinal results

© 2021 Wertz J et al. *JAMA Psychiatry.*

from the Berlin Aging Study. *Psychol Aging*. 2009;24(3):654-667. doi:10.1037/a0016510
19. Mock SE, Eibach RP. Aging attitudes moderate the effect of subjective age on psychological well-being: Evidence from a 10-year longitudinal study. *Psychol Aging*. 2011;26(4):979-986. doi:10.1037/a0023877
20. Belsky DW, Caspi A, Israel S, Blumenthal JA, Poulton R, Moffitt TE. Cardiorespiratory fitness and cognitive function in midlife: Neuroprotection or neuroselection? *Ann Neurol*. 2015;77(4):607-617. doi:10.1002/ana.24356
21. Belsky DW, Moffitt TE, Corcoran DL, et al. The genetics of success: How single-nucleotide polymorphisms associated with educational attainment relate to life-course development. *Psychol Sci*. 2016;27(7):957-972. doi:10.1177/0956797616643070
22. Belsky DW, Moffitt TE, Houts R, et al. Polygenic risk, rapid childhood growth, and the development of obesity: Evidence from a 4-decade longitudinal study. *Arch Pediatr Adolesc Med*. 2012;166(6):515-521. doi:10.1001/archpediatrics.2012.131
23. Williams S, Poulton R. Birth size, growth, and blood pressure between the ages of 7 and 26 years: Failure to support the fetal origins hypothesis. *Am J Epidemiol*. 2002;155(9):849-852. doi:10.1093/aje/155.9.849
24. Sears MR, Greene JM, Willan AR, et al. A longitudinal, population-based, cohort study of childhood asthma followed to adulthood. *N Engl J Med*. 2003;349(15):1414-1422. doi:10.1056/NEJMoa022363
25. Pronk M, Lissenberg-Witte BI, van der Aa HPA, et al. Longitudinal relationships between decline in speech-in-noise recognition ability and cognitive functioning: The longitudinal aging study amsterdam. *J Speech, Lang Hear Res*. 2019;62(4S):1167-1187. doi:10.1044/2018_JSLHR-H-ASCC7-18-0120
26. Dubno JR, Dirks DD, Morgan DE. Effects of age and mild hearing loss on speech recognition in noise. *J Acoust Soc Am*. 1984;76(1):87-96. doi:10.1121/1.391011
27. Cameron S, Dillon H. Development of the Listening in Spatialized Noise-Sentences Test (LISN-S). *Ear Hear*. 2007;28(2):196-211. doi:10.1097/AUD.0b013e318031267f
28. Noble W, Jensen NS, Naylor G, Bhullar N, Akeroyd MA. A short form of the Speech, Spatial and Qualities of Hearing scale suitable for clinical use: The SSQ12. *Int J Audiol*. 2013;52(6):409-412. doi:10.3109/14992027.2013.781278
29. Boothroyd A. Developments in speech audiometry. *Br J Audiol*. 1968;2(1):3-10. doi:10.3109/00381796809075436
30. Welch D, Dawes PJD. Variation in the normal hearing threshold predicts childhood IQ, linguistic, and behavioral outcomes. *Pediatr Res*. 2007;61(6):737-744. doi:10.1203/pdr.0b013e31805341c1
31. Nomura H, Ando F, Niino N, Shimokata H, Miyake Y. Age-related change in contrast sensitivity among Japanese adults. *Jpn J Ophthalmol*. 2003;47(3):299-303. doi:10.1016/S0021-5155(03)00011-X
32. Leat SJ, Legge GE, Bullimore MA. What is low vision? A re-evaluation of definitions. *Optom Vis Sci*. 1999;76(4):198-211. doi:10.5005/jp/books/10448_1
33. Marron JA, Bailey IL. Visual factors and orientation-mobility performance. *Optom Vis Sci*. 1982;59(5):413-426. doi:10.1097/00006324-198205000-00009
34. Frost NA, Sparrow JM, Durant JS, Donovan JL, Peters TJ, Brookes ST. Development of a questionnaire for measurement of vision-related quality of life. *Ophthalmic Epidemiol*. 1998;5(4):185-210. doi:10.1076/opep.5.4.185.4191
35. Wilson GA, Welch D. Does amblyopia have a functional impact? Findings from the

Dunedin Multidisciplinary Health and Development Study. *Clin Exp Ophthalmol*. 2013;41(2):127-134. doi:10.1111/j.1442-9071.2012.02842.x
36. Bohannon RW, Larkin PA, Cook AC, Gear J, Singer J. Decrease in timed balance test scores with aging. *Phys Ther*. 1984;64(7):1067-1070. doi:10.1093/ptj/64.7.1067
37. Hurvitz EA, Richardson JK, Werner RA, Ruhl AM, Dixon MR. Unipedal stance testing as an indicator of fall risk among older outpatients. *Arch Phys Med Rehabil*. 2000;81(5):587-591. doi:10.1016/S0003-9993(00)90039-X
38. Springer BA, Marin R, Cyhan T, Roberts H, Gill NW. Normative values for the unipedal stance test with eyes open and closed. *J Geriatr Phys Ther*. 2007;30(1):8-15. doi:10.1519/00139143-200704000-00003
39. Vereeck L, Wuyts F, Truijen S, Van de Heyning P. Clinical assessment of balance: Normative data, and gender and age effects. *Int J Audiol*. 2008;47(2):67-75. doi:10.1080/14992020701689688
40. Bayley N. *The Bayley Scale of Infant Development.* New York, NY: Psychological Corp; 1969.
41. Arnheim DD, Sinclair WA. *The Clumsy Child*. St Louis, MO: VC Mosby Co; 1974.
42. Verghese J, Holtzer R, Lipton RB, Wang C. Mobility stress test approach to predicting frailty, disability, and mortality in high-functioning older adults. *J Am Geriatr Soc*. 2012;60(10):1901-1905. doi:10.1111/j.1532-5415.2012.04145.x
43. The RAND Corporation. RAND 36-Item Short Form Survey (SF-36).
44. McCarthy D. *McCarthy Scales of Children's Abilities*. New York, NY: Psychological Corp; 1972.
45. Deary IJ, Corley J, Gow AJ, et al. Age-associated cognitive decline. *Br Med Bull*. 2009;92(1):135-152. doi:10.1093/bmb/ldp033
46. Marioni RE, Strachan MWJ, Reynolds RM, et al. Association between raised inflammatory markers and cognitive decline in elderly people with type 2 diabetes: The Edinburgh Type 2 Diabetes Study. *Diabetes*. 2010;59(3):710-713. doi:10.2337/db09-1163
47. Aichele S, Rabbitt P, Ghisletta P. Life span decrements in fluid intelligence and processing speed predict mortality risk. *Psychol Aging*. 2015;30(3):598-612. doi:10.1037/pag0000035
48. Wechsler D. *Wechsler Adult Intelligence Scale: WAIS-IV: Technical and Interpretive Manual*. Pearson; 2008.
49. Broadbent DE, Cooper PF, FitzGerald P, Parkes KR. The Cognitive Failures Questionnaire (CFQ) and its correlates. *Br J Clin Psychol*. 1982;21(1):1-16. doi:10.1111/j.2044-8260.1982.tb01421.x
50. Wechsler D. *Manual for the Wechsler Intelligence Scale for Children – Revised*. New York, NY: Psychological Corporation; 1974.
51. Caspi A, McClay J, Moffitt TE, et al. Role of genotype in the cycle of violence in maltreated children. *Science*. 2002;297(5582):851-854. doi:10.1126/science.1072290
52. Poulton R, Caspi A, Milne BJ, et al. Association between children's experience of socioeconomic disadvantage and adult health: a life-course study. *Lancet*. 2002;360(9346):1640-1645. doi:10.1016/S0140-6736(02)11602-3

© 2021 Wertz J et al. *JAMA Psychiatry*.
